# The Validity, Reliability, and Feasibility of Measurement Tools Used to Assess Sleep of Pre-school Aged Children: A Systematic Rapid Review

**DOI:** 10.3389/fped.2021.770262

**Published:** 2021-11-26

**Authors:** Sophie M. Phillips, Carolyn Summerbell, Helen L. Ball, Kathryn R. Hesketh, Sonia Saxena, Frances C. Hillier-Brown

**Affiliations:** ^1^Department of Sport and Exercise Sciences, Durham University, Durham City, United Kingdom; ^2^The Centre for Translational Research in Public Health (Fuse), Newcastle Upon Tyne, United Kingdom; ^3^Department of Anthropology, Infancy and Sleep Centre, Durham University, Durham City, United Kingdom; ^4^Department of Population Policy & Practice Research and Teaching, UCL Great Ormond Street Institute of Child Health, London, United Kingdom; ^5^Centre for Diet and Activity Research and MRC Epidemiology Unit, University of Cambridge, Cambridge, United Kingdom; ^6^School of Public Health, Imperial College London, London, United Kingdom; ^7^Population Health Sciences Institute, Newcastle University, Newcastle Upon Tyne, United Kingdom; ^8^Human Nutrition Research Centre, Newcastle University, Newcastle Upon Tyne, United Kingdom; ^9^Newcastle University Centre of Research Excellence in Healthier Lives, Newcastle University, Newcastle Upon Tyne, United Kingdom

**Keywords:** review, sleep, pre-school, measurement, movement, validity, reliability, feasibility

## Abstract

**Background:** Sleep of pre-school aged children is important for their health and development, but there are currently no standards for measuring sleep in this age group. We aimed to examine the validity, reliability and feasibility of tools used to assess sleep of pre-school aged children.

**Methods:** Studies were eligible for inclusion if they examined the validity and/or reliability and/or feasibility of a measurement tool used to examine sleep of pre-school aged children (aged 3–7 years). We systematically searched six electronic databases, grey literature and trial registries. We manually searched topic specific journals, reference and citations of included studies, and reference lists of existing reviews. We extracted data and conducted a risk of bias assessment on the included studies using the COnsensus-based Standards for the selection of health Measurement INstruments (COSMIN) risk of bias checklist. We used a narrative synthesis to present the results.

**Results:** Sixteen studies met the inclusion criteria: these explored accelerometers (*n* = 3) and parental reported tools (*n* = 13; nine questionnaires, six diaries). Studies assessed construct validity (*n* = 3), criterion validity (*n* = 1), convergent validity (*n* = 13), test-retest reliability (*n* = 2), internal consistency (*n* = 4) and feasibility (*n* = 12). Most studies assessed the convergent validity of questionnaires and diaries compared with accelerometers, but the validity of accelerometers for sleep in this age group is unknown. Of studies with a low risk of bias, one sleep diary was shown to be valid for measuring sleep duration. No measurement tools were appropriate for determining sleep quality. Reporting of reliability and feasibility was minimal.

**Discussion:** The evidence base in this field is limited, and most studies had high risk of bias. Future research on sleep in pre-school aged children should focus on assessing the validity, reliability and feasibility of accelerometers, which in turn will improve the quality of studies that assess questionnaires and diaries against accelerometers.

**Systematic Review Registration:** https://www.crd.york.ac.uk/prospero/display_record.php?ID=CRD42021230900; PROSPERO: CRD42021230900.

## Introduction

Sleep plays an essential role in the health and development of children in the early years (1–3), but adequate measurement tools are needed to appropriately assess parameters of sleep in young children. Previous paediatric sleep research has focused on the medical model of sleep, including the presence or absence of sleep disorders (4). More recently, studies have reported on the promotion of healthy sleep, including sufficient duration, quality and timing of sleep (2, 4–6); however, measurement of these parameters is less well established (7).

The World Health Organization (WHO) considered this emerging evidence base to be of sufficient interest to warrant inclusion of the importance of healthy sleep in children in their Report on Ending Childhood Obesity (8). This report suggested that the development of guidelines on sleep time, alongside sedentary behaviour (including screen time) and physical activity, was important for the appropriate growth and development of healthy habits of pre-school aged children. This recommendation was included in the subsequent 2019 WHO guidelines for pre-school aged children, which include guidelines for 24 h movement encompassing physical activity, sedentary behaviour and sleep (9). The WHO guidelines recommend that pre-school aged children should have 10–13 h of good quality sleep per 24 h day (9), based on the National Sleep Foundation's recommendations (10, 11).

Given the growing public health policy interest and potential impact on guidance of healthy sleep, there is a need for appropriate measurement and monitoring systems to gain a better understanding of sleep at population level (5), including practical ways of measuring sleep at scale. Measurement tools to assess parameters of healthy sleep are important for the advancement of research in this area, for policy makers to provide robust public health recommendations, and to update and improve national surveillance of sleep (12). Importantly, there is a need for measurement tools that examine sleep beyond solely determining whether sleep disorders and disturbances are present (7).

Sleep of pre-school aged children can be measured using various methods, including proxy reported measurement tools (questionnaires and diaries), device based measurement tools (accelerometers) and videosomnography. Alternative methods are primarily used to detect the physiological elements of sleep, based on brain activity, including polysomnography, electroencephalography and high-density electroencephalography (13, 14). Although polysomnography is classified as gold standard for determining sleep (15), questionnaires, diaries, and accelerometers are most frequently used within research studies (3) due to their practicality and ability to determine habitual sleep. The measurement tool selected should be related to the dimension of sleep being measured, alongside availability of resources (time, financial costs) and equipment (13). In addition, these measurement tools should have appropriate measurement properties, including being valid and reliable, alongside being feasible in the target population. Recent systematic reviews exploring associations between sleep and health indicators and other movement behaviours reported that very few studies used valid and reliable methods, with inconsistencies in the way in which tools were used to estimate sleep (3, 16).

Several existing reviews have provided an overview of available sleep assessment methods with information on measurement properties where available (7, 17–19) or have identified tools available for assessing parental knowledge of their child's sleep (20). Reviews examining the measurement properties of sleep measurement tools have focused on a specific type of tool, including accelerometers (21, 22), questionnaires (23, 24), or proxy reported tools (25, 26). However, to date, no review has examined the measurement properties of the full range of measurement tools that have been used to assess sleep, nor have they explored a range of measurement properties (validity, reliability and feasibility), and nor have they focused specifically on pre-school aged children. The aim of our review was to examine the validity, reliability, and feasibility of measurement tools used to examine the sleep of pre-school aged children in the general population. Our rationale was to focus on sleep measurement tools evaluated in children aged 3–7 years, to ensure inclusion of children who have not yet reached the age of formal schooling internationally (27).

## Methods

We conducted a rapid review (28–30) in line with Preferred Reporting Items for Systematic Reviews and Meta-Analyses (PRISMA) criteria (31) (Supplementary File 1). We registered our protocol with the PROSPERO database (CRD42021230900) and followed the AMSTAR protocol, an assessment tool for quality assurance of systematic reviews, as closely as possible (32, 33).

### Search Strategy

We systematically searched six major electronic databases [Scopus (Science Direct) Web of Science (Web of Science), MEDLINE (Ovid), APA PsycARTICLES (EBSCOhost), APA PsycINFO (EBSCOhost), and SPORTDiscus (EBSCOhost)], the grey literature (opengrey.eu) and trial registries (International Clinical Trials Registry Platform) in April 2021 to identify relevant studies. For completeness, we manually searched topic specific journals (*Sleep* and *Sleep Health*), reference and citations of the included studies, and references of selected existing reviews (7, 17–26) on 4th May 2021.

The search strategy included combinations of the construct (sleep); population (pre-school, early years, early childhood, young children and kindergarten); and measurement properties (assessment, measurement, method, valid, reliable, feasible). Searches were adapted to each database, alongside the use of appropriate Boolean operators and database specific filters (Supplementary File 2). No restrictions were placed on language or year of study. We conducted multiple preliminary searches to ensure that the search strategy could identify a selection of clearly eligible studies (34–37).

### Eligibility Criteria

Articles were eligible for inclusion if their aim was to examine the measurement properties (validity and/or reliability and/or feasibility) of a tool used to measure the sleep of pre-school children aged 3–7 years old. There were no restrictions on study design or setting. Only full text articles or abstracts where sufficient information was available were included. Table 1 provides an overview of the definitions of measurement properties examined in this review (38–41).

**Table 1:** Definition of each of the measurement properties included in this review.

**Measurement property**	**Definition**
**Validity**	*Ability for a measure to accurately reflect the construct it is designed to measure*.
Construct (or structural) validity	The extent to which the measurement tool actually tests the hypothesis or theory they are measuring. This is usually examined by just the one measurement tool being explored, using statistical methods such as confirmatory or exploratory factor analysis.
Content validity	Extent to which a measure covers all aspects of the intended domains or dimensions that it claims to measure. This is usually examined through qualitative means. Often includes assessment of **face validity**, which is the appearance of a measure, in that it appears to measure what it claims to measure.
Criterion validity	Output of a measure produces similar results to a “gold standard”. This includes studies that have examined a tool against polysomnography (which may be alongside videosomnography or direct observation).
Convergent (concurrent) validity	The extent of the agreement between measures. This includes studies that have examined comparisons between multiple measurement tools (such as accelerometers, diaries and questionnaires) but do not include a criterion method of polysomnography or videosomnography.
**Reliability**	*Extent to which a tool gives measurements that are consistent, stable and repeatable*.
Test-retest reliability	The extent to which a measure can obtain similar results in repeated trials, keeping as many conditions stable as possible.
Internal consistency	The extent to which items among a measurement tool that propose to measure the same construct are interrelated.
**Feasibility**	The extent to which a measurement tool: is suitable for the target population; can be successfully delivered in the target population/context; shows promise of being successful within the intended population. Includes participant and researcher acceptability, and cost, which can be assessed for all measurement tools through qualitative feedback of participants and through missing or lost data occurred from the measurement tool.

*Definitions guided by: (38–41)*.

Articles were excluded if:

a) The measurement tool was examined in children outside of the pre-school age range (aged <3 or >7 years old), and did not include independent analysis of children within this age group (e.g., did not include an analysis of the measurement tool for 3–5 year olds only).
b) The measurement tool was examined in children with clinically diagnosed conditions that may impact sleep (e.g., autism, attention deficit hyperactivity disorder (ADHD), cerebral palsy, sleep apnoea).
c) The measurement tool had a primary purpose of determining clinical sleep problems and disturbances, such as sleep apnoea or sleep disordered breathing.
d) The article was a book or review.

### Screening for Relevant Studies

All identified articles were imported into a referencing manager software (Endnote X20) and duplicates removed. Titles and abstracts of identified articles were screened, followed by full texts of potentially relevant articles, by the lead author (SMP) for inclusion. Articles where the eligibility was uncertain were independently double screened by a second author (FCHB); consensus on the eligibility of these articles was reached through discussion. Where eligibility was unclear, authors of the studies were contacted to ensure appropriate inclusion or exclusion of the study (*n* = 4).

### Data Extraction

We used a pre-piloted data extraction form to extract data from the included studies. Data from all relevant studies was extracted by the lead author (SMP) and checked for accuracy by a second author (CS). Extracted information included: study characteristics (authors, country, publication year, sample size); participant characteristics (age, sex, ethnicity, socioeconomic profile); aims of the study; methods used, including: measurement tool examined (e.g., sleep diary), comparison measurement tool(s), study setting (free living/laboratory), number of nights of data collected and used in analysis, whether week nights and weekend nights were calculated separately, 24 h sleep time or night time sleep only, whether data were systematically collected on day time naps, and for device based measurement tools: type, placement, epoch, cut point and algorithms used; outcome measures explored (e.g., sleep duration); measurement property examined (e.g., convergent validity); statistical method used; results of the study (including information on missing data) and sources of funding.

### Risk of Bias of Included Studies

Risk of bias assessment of all included studies was conducted independently by the lead review author (SMP), and double checked by a second author (CS). We conducted a risk of bias assessment on the included studies using the COnsensus-based Standards for the selection of health Measurement INstruments (COSMIN) risk of bias checklist (40, 42, 43). Based on the studies included in our review we conducted the assessment using the sub-sections relating to structural (construct) validity, criterion validity, construct (convergent) validity, test-retest reliability and internal consistency. Only those sections relevant to the particular study were conducted. Each item was scored using the four point scale outlined by COSMIN (very good, adequate, doubtful, inadequate) (43). The overall quality assessment of a study was determined using “the worst score counts” principle (e.g., if one item was scored as “inadequate”, the overall score of the measurement property in that study would be “inadequate”) (44). Risk of bias may be present if the overall quality assessment is doubtful or inadequate, or if there is only one study where the risk of bias is quality assessed as adequate (42).

### Interpretation and Synthesis of the Results

To ensure consistency in the interpretation of the statistical results of individual studies, we predefined scores and provide an overview of what constitutes a “weak”, “moderate”, or “good” statistical result for the measurement properties of validity or reliability in Table 2 (39, 45–48). Feasibility was interpreted narratively based on the type of assessment used within each study.

**Table 2:** Main statistical analyses and interpretation of statistics.

**Statistical Analysis**		**Weak**	**Moderate**	**Good**
Correlations (r)	Pearson's	<0.60	0.60–0.79	≥0.80
	Spearman's	<0.60	0.60–0.79	≥0.80
	Polyserial correlation coefficient	<0.60	0.60–0.79	≥0.80
	Intraclass correlation coefficient	<0.60	0.60–0.69	≥0.70
Cronbach's alpha		<0.60	0.60–0.69	≥0.70
Bland Altman^a^		>31 min deemed not satisfactory agreement		≤ 30 min
*T*-tests		b	b	b
Feasibility		Narrative description		

*Interpretation in line with: (39, 45–48)*.

a *Extracted based on the interpretation of the included studies using this analysis method, a priori defined satisfactory agreement of ≤ 30 minutes only*.

b *Depends on the units of measure*.

## Results

### Study Selection

**Figure 1:**
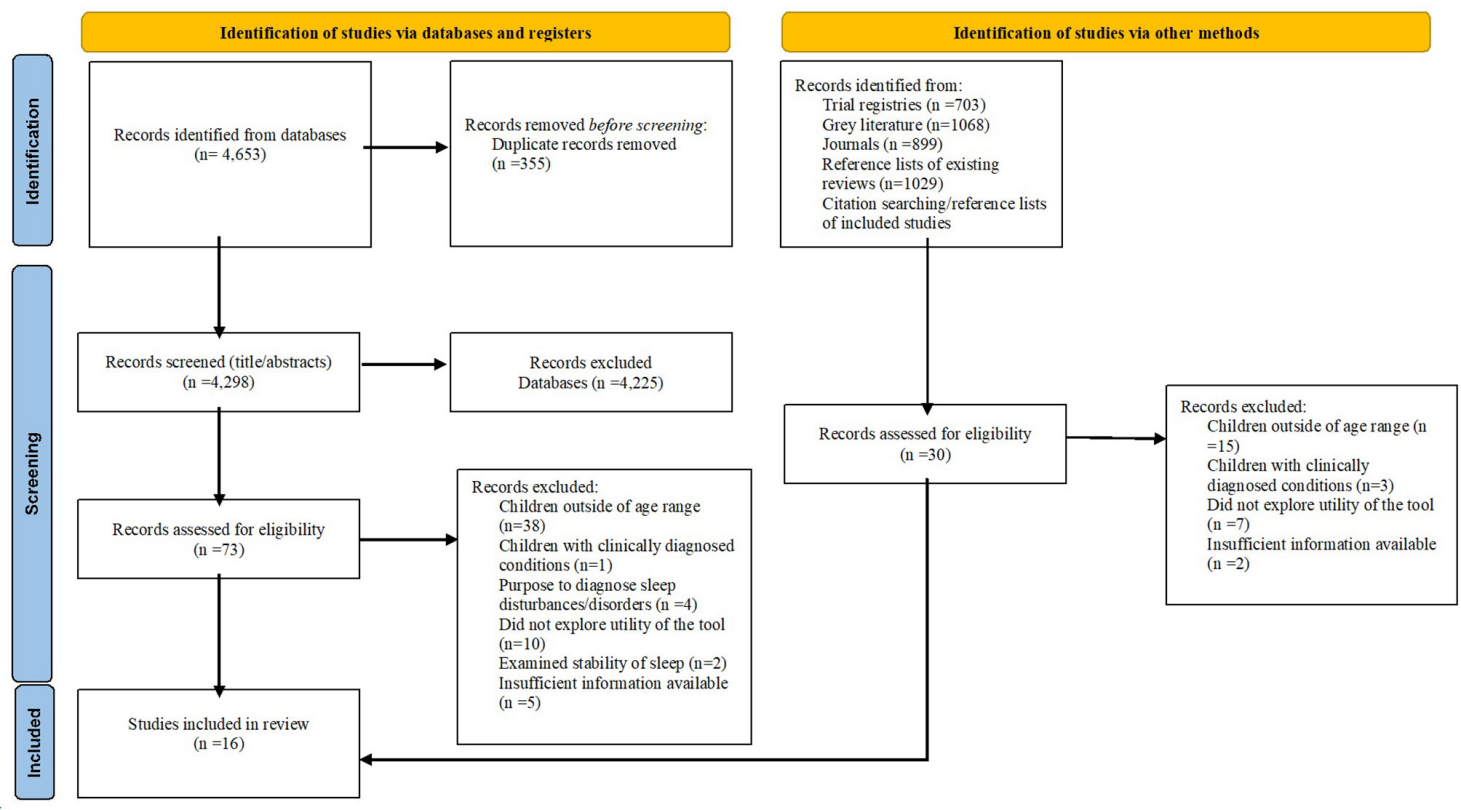
PRISMA flow chart of included studies (31).

Initial database searches identified 4,298 articles. From this 73 full text articles were screened and, of these, 13 articles met the eligibility criteria and were included in the review. A further three articles were identified through searching of reference lists of included studies and were included in the review (Figure 1). Studies were mainly excluded due to including children outside of the specified age range. Excluded studies with reasons are outlined in Supplementary File 3.

### Study Characteristics

Included articles were published between 2001 and 2021, and were all conducted in high income countries (49): USA (*n* = 5), Japan (*n* = 3), Israel (*n* = 2), China (*n* = 1), New Zealand (*n* = 1), Portugal (*n* = 1), Spain (*n* = 1), Switzerland (*n* = 1) and UK (*n* = 1). Sample sizes ranged from 14 (50) to 346 children (51). All studies that reported the sex of the children included both male and female children. The median age of the included children was 4.9 years. Most studies examined the measurement properties of the tools in free-living conditions to determine habitual sleep behaviours (*n* = 15). One study was laboratory based and used polysomnography as the criterion method to measure sleep (50).

Thirteen studies examined parental reported measurement tools (questionnaires and diaries), and three remaining studies examined accelerometers. Fourteen studies examined the measurement properties of one measurement tool only and two studies examined the measurement properties of two measurement tools (35, 37). Nine studies examined questionnaires; two studies generated a new questionnaire (36, 37), three adapted questionnaires developed for other age groups (35, 51, 52), and four studies used the *Children's Sleep Habits Questionnaire* (CSHQ) (two in English, one variation translated into Spanish and one in Chinese), a measure originally developed for children aged 4–10 years old (34, 53–55). Six studies examined the measurement properties of different diaries. Diaries were specific to the study, with two studies utilising parental diaries frequently used within a clinical care setting (37, 56). Three studies examined the measurement properties of three types of accelerometer: Actigraph GT3X+, Fitbit Ultra and MicroMini Motionlogger.

The most frequently reported measurement property was convergent validity (*n* = 13). Three studies examined the construct (structural) validity of the tools, one examined criterion validity, two studies the test-retest reliability and four the internal consistency. No studies reported the qualitative feasibility of the tools, though twelve studies reported missing data or non-completion that demonstrated an element of feasibility. Table 3 provides an overview of each study and the measurement properties that the study examined.

**Table 3:** Overview of measurement properties examined in each study.

**Study**	**Country**	**Measurement instrument under study**	**Construct validity**	**Criterion validity**	**Convergent validity**	**Test-retest reliability**	**Internal consistency**	**Feasibility**
**Accelerometers**								
Meltzer et al. (50)	USA	Fitbit Ultra		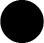				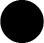
Tracy et al. (57)	USA	Actigraph GT3X+			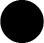			
Staples et al. (58)	USA	MicroMini Motionlogger	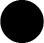				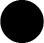	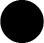
**Questionnaires**								
Duraccio et al. (34)	USA	Children's Sleep Habits Questionnaire (CSHQ)			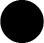			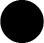 ^a^
Perpétuo et al. (54)	Portugal	Children's Sleep Habits Questionnaire (CSHQ)			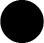			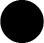
Lucas de la Cruz et al. (53)	Spain	Child Sleep Habits Questionnaire (CSHQ- Spanish)	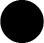		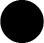	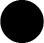	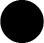	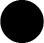
Tan et al. (55)	China	Children's Sleep Habits Questionnaire (CSHQ- Chinese)	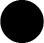				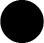	
Ishihara et al. (51)	Japan	Children's ChronoType Questionnaire (CCTQ- Japanese)			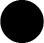	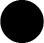	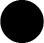	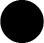
Iwasaki et al. (35)	Japan	Brief 12 item questionnaire			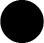			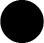 ^a^
Kushnir and Sadeh, (52)	Israel	Brief Child Sleep Questionnaire (BCSQ)			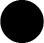			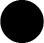 ^a^
Sekine et al. (36)	Japan	Sleep habits questionnaire			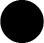			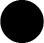 ^a^
Werner et al. (37)	Switzerland	Sleep schedule time questionnaire (SSTQ)			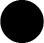			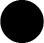 ^a^
**Diaries**								
Galland et al. (59)	New Zealand	Sleep diary			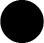			
Iwasaki et al. (35)	Japan	Sleep diary			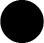			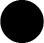 ^a^
Jones and Ball, (60)	UK	Sleep diary			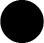			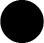 ^a^
Lam et al. (56)	USA	Sleep diary			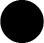			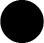 ^a^
Tikotzy and Sadeh, (61)	Israel	Sleep diary			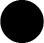			
Werner et al. (37)	Switzerland	Sleep diary			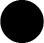			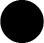 ^a^

a *Feasibility assessment for comparison tool only*.

### Risk of Bias

Three studies assessing construct (structural) validity were quality assessed as adequate (55, 58) or doubtful (53). One study assessing criterion validity was quality assessed as inadequate (50) due to the type of analysis conducted. The majority of studies assessing the convergent validity of the tools were quality assessed as high risk of bias; eight inadequate (34, 35, 37, 51–54, 56) and four doubtful (36, 57, 59, 60), with the exception of one study quality assessed as very good (61). Main reasons for poor methodological quality of the studies was due to unknown measurement properties of the comparator tools. Additionally, in some studies the measurement tools were assessing different time periods [e.g., questionnaire measuring the week before the period of accelerometer wearing (34, 52)]. Two studies examining test-retest reliability were quality assessed as inadequate (51, 53) due to the choice of statistical method used. Four studies examining internal consistency were quality assessed as very good (51, 53, 55, 58). The full risk of bias assessment can be found in Supplementary File 4. No studies were removed from the overall analysis based on the risk of bias assessment, however, the quality of studies is acknowledged throughout the results and discussion.

### Summary of Measurement Properties of Measurement Tools

Table 4 provides a summary table of the evidence for validity (criterion and convergent) and reliability of the measurement tools. Construct validity and feasibility are not included in this table due to not being able to present the results of these studies in this way. Detailed information of studies for each measurement property can be found in Supplementary File 5 (construct validity), Supplementary File 6 (criterion validity), Supplementary File 7 (convergent validity), Supplementary File 8 (reliability), and Supplementary File 9 (feasibility).

**Table 4:** Summary table of the rating^*^ of validity (criterion and convergent) and reliability (test-retest and internal consistency), and quality (risk of bias^**^), of the measurement tools for sleep parameters of pre-school aged children.

**Measurement instrument under study**	**Validity**							**Reliability**		**Risk of bias assessment** **(Inadequate, Doubtful,** **Adequate, or** **Very good)**	**Reference to the included studies**
	**Sleep duration**	**Sleep onset time**	**Bedtime**	**Sleep end/wake up time**	**Night wakings**	**Sleep latency**	**Nap time**	**Test-retest reliability**	**Internal consistency**		
**Criterion validity**											
**Accelerometers**											
Fitbit Ultra (normal mode)	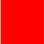				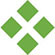					Inadequate	(50)
**Convergent validity**											
**Accelerometers**											
Actigraph GT3X+	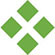									Doubtful	(57)
MicroMini Motionlogger									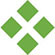	Very good	(58)
**Questionnaires**											
Children's sleep habits questionnaire (English)	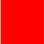				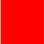	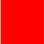				Inadequate	(34)
Children's sleep habits questionnaire (English)	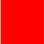			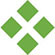	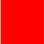					Inadequate	(54)
Children's sleep habits questionnaire (Spanish)	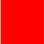				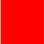	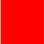		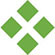 ^a^	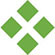	Inadequate (convergent and test-retest)	(53)
										Very good (internal consistency)	
Children's Sleep Habits Questionnaire (Chinese)									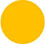	Very good	(55)
Children's ChronoType	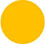 ^b^	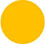	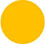	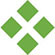 ^b^		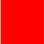		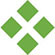	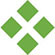	Inadequate (convergent and test-retest)	(51)
Questionnaire (Japanese)	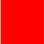 ^c^			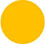 ^c^						Very good (internal consistency)	
Brief 12 item questionnaire	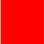	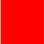	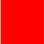	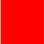	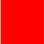	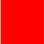				Inadequate	(35)
Brief Child Sleep Questionnaire	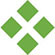	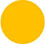			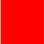					Inadequate	(52)
Sleep habits questionnaire	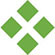									Doubtful	(36)
Sleep schedule time questionnaire	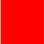	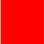		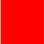						Inadequate	(37)
**Diaries**											
Sleep diary (5 min intervals)							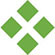			Doubtful	(59)
Sleep diary (daily information)	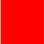 ^b^	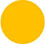 ^b^		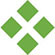 ^b^	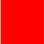					Inadequate	(35)
	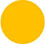 ^c^	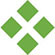 ^c^		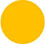 ^c^							
Sleep diary (daily information)		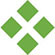		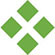			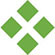			Doubtful	(60)
Sleep diary (daily information)	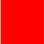	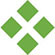		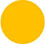	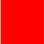		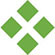			Inadequate	(56)
Sleep diary (daily information)	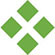	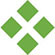		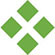	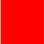	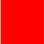				Very good	(61)
Sleep diary (15 min intervals)	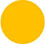	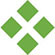		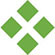	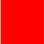					Inadequate	(37)

* *The methods used to assess the **rating of the measurement tool** are based on the interpretation of statistics detailed in Table 2 and are indicated in this summary table as: Good, 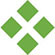 Moderate, 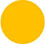 Weak, 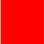. These ratings do not include the risk of bias assessment; this is included in the final column to demonstrate the quality of the evidence of each study. Ratings of measurement tools assessed as low risk of bias are highlighted in the blue cells*.

** ***Risk of bias*** *may be present if the overall quality assessment is doubtful or inadequate, or if there is only one study where the risk of bias is quality assessed as adequate*.

a *Representative of all subscales apart from night awakenings and daytime sleepiness which had weak ratings*;

b * Weekdays*;

c * Weekend days*.

*This table shows a summary of the results of studies where they assessed the criterion validity (in comparison with polysomnography), convergent validity (in comparison with accelerometry), test-retest reliability and internal consistency of the tools. This table does not include results on the construct (structural) validity or feasibility of the tools, due to the nature of these studies resulting in the information not being able to be presented in such a way. Please refer to the text for a narrative overview of studies that assessed these measurement properties*.

### Validity

#### Construct (Structural) Validity

Two studies examined the construct validity of the CSHQ: one a Spanish translation (53) and a Chinese translation (55). Lucas-de la Cruz and colleagues conducted an exploratory factor analysis and deemed keeping the same factor structure as the original questionnaire to be appropriate (62). Tan and colleagues conducted a confirmatory factor analysis and determined that no existing factor structures were suitable for the data with pre-school aged children. Following this, they performed an exploratory and a further confirmatory factor analysis to determine a new four factor structure, including: bedtime behaviours, sleep behaviours, morning waking, and daytime sleepiness (55).

One study examined the construct validity of the outcome measures of the MicroMini Motionlogger accelerometer using principal component analysis (58). This study revealed a four component structure: sleep activity, sleep variability, sleep timing and sleep duration, which could categorise accelerometer outcome variables. Daytime sleep and sleep latency represented exclusive elements of sleep that could not be categorised into factor structures.

#### Criterion Validity

One study examined the criterion validity of the Fitbit Ultra compared with polysomnography (50). The Fitbit Ultra (sensitive mode) underestimated sleep time and sleep efficiency, and overestimated wake after sleep onset. The Fitbit Ultra (normal mode) overestimated sleep time and sleep efficiency, but there was no significant difference for wake after sleep onset.

#### Convergent Validity

##### Accelerometers

One study examined the ability of the Actigraph GT3X+ accelerometer to determine bedrest and wake time, using a newly developed decision tree algorithm specific to pre-school aged children (57). The developed algorithm was able to detect bedrest and wake time similarly to visual identification of the data and was better at identifying bedrest than algorithms developed in other age groups that are often applied to young children (63, 64). There were significant differences between the outcomes of the algorithm and parental report (p < 0.001).

##### Questionnaires

Eight studies examined the convergent validity of six different questionnaires (all parental reported). The most frequently assessed questionnaire was the CSHQ, in English (34, 54) and Spanish (53). The majority of questionnaires assessed “typical” sleep (34, 37, 53–55), others varied including: daily reporting (36), past week (52) and the past month (35). Three questionnaires systematically collected data on naps (34, 37, 54), however none reported on this outcome measure. Four studies examined weekday and weekend days separately (35, 37, 51, 54), one study stated that data were collected on weekdays only (34).

All studies used an accelerometer as the comparison tool, with only two studies using the same accelerometer (*Actiwatch 2)* (51, 54). Two studies also used sleep diaries alongside accelerometers, to determine sleep onset and offset (51, 53) and average bedtime and wake time (34).

All questionnaires assessed sleep duration. The *Brief Child Sleep Questionnaire* (BCSQ) (52) and *sleep habits questionnaire* (36) showed high convergence with accelerometers for determining sleep duration (*r* = 0.85–*r* = 0.90). The *Children's ChronoType Questionnaire* (CCTQ) showed a moderate correlation for weekdays but weak for weekend days (51). The remaining questionnaires generally showed weak associations with accelerometry for determining sleep duration (34, 35, 37, 53, 54). No questionnaires were deemed satisfactory at determining sleep latency (34, 35, 51, 53) or night awakenings (34, 35, 52–54).

Results for wake up time were variable; the CSHQ was highly convergent with accelerometry more so for weekdays (7 min difference) than weekend days (28 min difference) (54), with similar patterns observed for the CCTQ (51). The *Sleep Schedule Time Questionnaire* (SSTQ) and *brief questionnaire* were deemed to be weak at determining wake up time (35, 37).

Bedtime reports were moderate for the CCTQ (51) and weak for the *brief questionnaire*, for weekday and weekend days (35). Sleep onset time reports were moderate for the CCTQ and BCSQ (51, 52) and weak for the *brief questionnaire* (35) and SSTQ (37).

##### Diaries

Six studies examined the convergent validity of sleep diaries. The format of the diaries varied, some used time intervals to determine sleep/wake status (56, 59), whilst others requested certain information, such as sleep onset and end time (35, 56, 60, 61). All diaries were parental reported, however, three studies explicitly stated that day time sleep records were based on information from nursery teachers (35, 56, 59).

Diaries were completed daily in all studies (35, 37, 56, 59–61), for a duration of 2 (59) to 6–8 days (37). The majority of diaries collected data on nap times (37, 56, 59–61), however only three reported results on this outcome measure (56, 59, 60). Three studies reported results for weekday and weekend days separately (35, 37, 56).

All studies used an accelerometer as the comparison tool; different types of accelerometers were used, although, it was uncertain whether two studies used the same accelerometer as reporting was unclear (35, 61). Diary and accelerometer data were collected simultaneously in all studies.

Three studies demonstrated that the sleep diaries were highly convergent for determining nap times in comparison with accelerometry (56, 59, 60). Diaries were generally similar to accelerometry for determining sleep onset (35, 37, 56, 60, 61) and sleep end/wake time (35, 37, 60, 61), with the exception of one study where parents reported that the child rose later than that detected by the accelerometer (56). Mixed results were reported for the diaries association with accelerometry for sleep duration, including: good (61), moderate (37), and weak (56). One diary showed weak correlations for weekday but moderate for weekend days (35). Similar to questionnaires, diaries were all rated as weak for determining night awakenings (35, 37, 56, 61). One study reported weak associations between the diary and accelerometer for sleep latency and sleep quality (based on sleep efficiency, true sleep time and night awakenings) (61).

### Reliability

#### Test-Retest

Two studies assessed the test-retest reliability of two different questionnaires (51, 53). The CCTQ (Japanese version) completed twice two weeks apart and the CSHQ (Spanish version) completed twice within three weeks. The questionnaires showed high correlations between administrations; CCTQ (*r* = 0.90) (51) and the CSHQ, particularly for sleep duration, (*r* = 0.81). Night awakenings showed weak test-retest (*r* = 0.56) (53).

#### Internal Consistency

Three studies examined the internal consistency of the questionnaires, results showed: moderate for the CSHQ (Chinese version) (α = 0.67) (55) and high for the CSHQ (Spanish version) (α = 0.81) (53) and CCTQ (Japanese version) (α = 0.77) (51).

The newly devised four factor structure for the MicroMini Motionlogger accelerometer (sleep activity, sleep variability, sleep timing and sleep duration, which could categorise accelerometer outcome variables) were determined to have high internal consistency (ranging from α = 0.89 to 0.95) (58).

### Feasibility

No studies explicitly examined the feasibility of the measurement tools through qualitative research. However, as per previous reviews (39), we included missing data, non-completion and other indicators of feasibility of the measure (such as completion time).

Twelve of the studies reported information that provided an indication on the feasibility of the measurement tools. This primarily consisted of missing data from the accelerometer, either when used as the comparison tool (34, 36, 37, 51–54, 56, 60) or tool under study (50, 58) for reasons including: technical problems, refusal to wear the device, or a lack of available valid data. One study reported that although the accelerometers were tolerated by the children, the cost of such devices (and associated licenced software required) was expensive (35).

One study reported that completion of the CSHQ (Spanish version) took 4–6 min (53). Several studies reported missing data from questionnaires either through non-completion or the questionnaire not being completed correctly (51, 53, 54).

### Generalisability of Results

#### Ethnicity

Studies that reported the ethnicity of the included children had samples who were predominantly Caucasian (34, 37, 59, 60), Hispanic/Latino (57), Chinese (55), and African American children (56).

#### Socioeconomic Profile

Studies that reported the socioeconomic profile of participants described the families of the children as mainly middle-upper class (34, 37, 60, 61), with the exception of one study that reported that children lived in areas within “mid-range” of deprivation (59). Two studies reported the educational level and working hours of parents of the included children (54, 55). One study reported no systematic differences in the outcomes of the diary and accelerometry between families in low and high socioeconomic groups (60).

For readers interested in studies limited to pre-school children aged 3–5 years old only, we provide a sub-analysis outlining full results for this age group in Supplementary File 10.

## Discussion

### Summary of Main Findings

This review is the first to examine the measurement properties of tools used to assess sleep of pre-school aged children. The majority of studies (13/16) that met the inclusion criteria examined questionnaires and diaries, with minimal studies (3/16) on the validity of accelerometry to measure sleep in pre-school aged children. The limited evidence base is congruent with previous research reporting that the measurement properties of sleep measurement tools are often not assessed (18–20, 23). Previous development and evaluation of measurement tools used to assess sleep of children has predominantly focused on children aged 6 years and older (7) and, therefore, studies assessing sleep in pre-school aged children, rarely use valid and reliable methods (3, 16). The conclusions made from research studies implementing tools with either poor or unknown measurement properties may therefore be biased and invalid (19).

Most studies included in the review assessed the measurement properties of questionnaires and diaries, primarily though convergent validity, using accelerometers as the comparison measure. Only one questionnaire (CSHQ) was assessed in multiple studies. There were very few studies that assessed the ability of the accelerometers to detect sleep specifically in the pre-school age range, with only one study examining the criterion validity of the Fitbit Ultra (50). The Fitbit Ultra was not used as a comparison tool in any other study. This raises concerns about the results of studies when the measurement properties and accuracy of the tool being used as the comparison are unknown. It is critical that accelerometers are validated against a criterion method of polysomnography and/or videosomnography in the pre-school population prior to accelerometers being used as a comparison tool for validating further tools. This would require expensive and intensive research methodology, which may not always be feasible.

Overall, based on the current very limited evidence base, the “*subjective daily information”* reported in the sleep diary proposed by Tikotzky and Sadeh (61) appeared most accurate for assessing sleep duration, and was based on a study with low risk of bias. However, this conclusion is from the results of one study only. The *Sleep Habits Questionnaire*, BCSQ and CCTQ were concordant with accelerometry for assessing sleep duration, with the CCTQ also demonstrating good reliability (51). However, these studies reported night time sleep only, were based on the results of one study each, and were determined to have high risk of bias. There were three diaries that directly assessed the outcome of day time sleep, all of which showed good accuracy (56, 59, 60).

All measurement tools assessed for the outcomes of sleep latency, night awakenings or wake after sleep onset were shown to be poor at determining these factors, with the exception of the Fitbit Ultra for measuring wake after sleep onset, using the normal mode only (50). This suggests that, at present, there is insufficient evidence to provide a conclusion on which measurement tools would be applicable to determine the sleep quality of pre-school aged children. Parental reported tools showing poor accuracy for determining night awakenings of young children has been highlighted previously (26). Suggested reasons for this include that children of this age may stop signalling their parents if they wake during the night (52) and also that accelerometers (used as comparison methods) overestimate night awakening (65, 66). This potential bias is important when inferring the accuracy of parental reported tools.

At present, given the limited and low quality evidence, we do not feel there is a questionnaire or accelerometer that could be recommended. However, if such methods are to be used the *BCSQ* (52) and *Sleep Habits Questionnaire* (36) show most promising results for the assessment of sleep duration of pre-school aged children. The Fitbit Ultra shows reasonable results for determining night wakings (50). However, the low quality of this evidence must be acknowledged.

Sleep onset and end time consistently showed higher convergence when reported from the diaries (35, 37, 60, 61) than questionnaires (35, 37, 52, 54). This is unsurprising given that in most instances diaries and accelerometry were being compared simultaneously, whilst questionnaires and accelerometers were not reflective of the same time frame. For example, questionnaires were measuring the week before the period of accelerometer wearing (34, 52), or the questionnaire was measuring “typical” sleep, whilst the accelerometer was measuring “in the moment” sleep (37, 53, 54). Research has demonstrated that the sleep patterns of pre-school age children vary even within a single week (67, 68). As such, when comparing measurement tools it would be important to ensure they are representative of the same time frame—as the fluctuations and variations in sleep may be incorrectly attributed to the measurement tool being less accurate.

Previous research has suggested that reporting of sleep duration may be more accurate for weekday nights than weekend nights (24). There were differences in accuracy of reporting between weekday and weekend days in this review (35, 51), but there were no consistent patterns in terms of which days were more accurately reported. Although the reasons for this are unknown, the differences highlight the importance of assessing both weekday and weekend day sleep in research.

Accelerometers were used as the comparison tool in the majority of the studies. However, the types of accelerometer, placement, epochs, algorithms and procedures to detect sleep and wake varied between studies. The algorithms used to assess sleep parameters when using accelerometers (34, 35, 52, 53, 56) were based on algorithms devised for adolescent and adult samples (63, 69), despite known differences in the sleep of individuals of different ages (70). This review found that a pre-school specific algorithm was more accurate at detecting bedrest and wake time in comparison with visual identification of the data, and outcomes differed, when compared with existing algorithms frequently used (57). This highlights the importance of the data processing decisions when using accelerometers to detect sleep of pre-school aged children, and the need for device and age specific algorithms to improve accuracy.

Diaries were often used alongside accelerometers to indicate at least bed and wake time. This is common practice, as accelerometers cannot distinguish sleep from other low energy behaviours such as sedentary behaviour (14, 21, 71). The use of a diary alongside an accelerometer has been shown to increase accuracy for measuring sleep (72). Accelerometers infer sleep based on the absence of movement, rather than being a direct measure of sleep (14). Additionally, accelerometer output data is heavily reliant on subjective data interpretation choices (73). As such, arguably, accelerometry should not be defined as an “objective” measure of sleep, as is often the case within the literature.

There is limited research on the feasibility of measurement tools used to assess sleep of pre-school aged children. There were few reports on the feasibility of the measurement tools and no studies directly assessed feasibility qualitatively. Measurement tools are only applicable for use when feasible in the population in which they are to be used, as such, feasibility should be given as much attention as validity and reliability during tool development and evaluation. The majority of included studies reported missing data for the accelerometer, either when this was the tool under study or comparison tool. This is important feasibility information to note, as device malfunction can result in whole datasets being disregarded (74). Future research should ensure that feasibility is assessed. In particular, more qualitative work exploring the acceptability and feasibility of measurement tools is warranted to understand perceptions of the tools.

Further, the content validity of the included tools was unknown as no studies commented on the development process of the proxy reported tools (40). Further qualitative research with parents and carers of pre-school aged children during development of proxy report based measurement tools is recommended to ensure the items of the tool, and the tools themselves, are relevant and comprehensive to the construct, population and context of use (40).

Disparities in sleep behaviours of young children based on ethnicity, income, and mother's level of education have been noted (75). Recent research has shown that parents of Hispanic children were more accurate at estimating their child's sleep duration and wake time than parents of White children (76). This may be explained by different sleep habits (e.g., higher rates of bed sharing in the Hispanic families) (76). This demonstrates the importance of ensuring tools are developed and evaluated with the population of interest. Additionally, studies included in this review were all conducted in high income countries, it is important for sleep measurement research with pre-school aged children in lower and middle income countries (24).

### Limitations of the Review

The main study limitation is the potential bias introduced by having only one reviewer to screen the studies, and conduct the data extraction and risk of bias of included studies (30). This reviewer is experienced in conducting reviews of this nature and any uncertainty on study eligibility was resolved through consultation with a second reviewer. Further, a second reviewer checked the outcomes of the data extraction and risk of bias assessment against the original studies.

Children's sleeping patterns change rapidly in the early years of life (10, 11). Although we included a broad age range in the current review to ensure inclusion of children who have not yet reached the age of formal schooling internationally (27), only two studies included children aged 7 in their samples (37, 53).

It was not possible to conduct a meta-analysis on the included studies due to the heterogeneity of measurement tools being examined and comparison measurement tools used, and the lack of multiple studies assessing the same measurement tool.

### Implications and Recommendations for Future Research

This review has important implications for the measurement of sleep moving forward, particularly due to the presence of sleep in public health discourse and in global recommendations for pre-school aged children (9). Quality tools with known measurement properties are needed both to develop an appropriate evidence base and to effectively monitor and evaluate sleep at population level. This review highlights clear gaps that must be addressed including:

1. Evaluation of the validity, reliability, and feasibility of accelerometry for the use of sleep measurement in pre-school aged children, including the data processing decisions, age and device specific algorithms, and placement. There is a particular need for validation against criterion methods including polysomnography and videosomnography.
2. Qualitative feasibility of measurement tools used to assess sleep of pre-school aged children, to ensure acceptability.

Additionally, the measurement of physical activity and sedentary behaviour has been explored independently from sleep, with researchers in the separate fields advancing the same technology over years (77). There is now more interest in assessing the whole 24 h of the day, and the movement behaviours that this encompasses from sleep to physical activity (77, 78), and therefore, scope to bring these fields together when exploring measurement of these behaviours moving forward.

## Conclusion

This review highlights the scarcity of evidence exploring the measurement properties of tools used to examine the sleep of pre-school aged children and some clear gaps in knowledge. There is a need for further evaluation of measurement tools used to assess the sleep of pre-school aged children. In particular, evaluation of the validity and reliability of accelerometers, to improve the quality of studies assessing measurement properties of questionnaires and diaries, as well as assessing the qualitative feasibility of all measurement tools.

## Data Availability Statement

The original contributions presented in the study are included in the article/Supplementary Material, further inquiries can be directed to the corresponding author/s.

## Author Contributions

SP was involved in the conception, design, data screening, data extraction, risk of bias, data synthesis, interpretation, write up of the manuscript, and initially drafted the article. CS was involved in the conception, design, data extraction, risk of bias, interpretation, and write up of the manuscript. FH-B was involved in the conception, design, study eligibility, interpretation, and write up of the manuscript. HB, KH, and SS were involved in the conception, design, interpretation, and write up of the manuscript. All authors contributed to subsequent drafts, approved the final manuscript, and have approved the submitted version.

## Funding

This review was funded and supported by the National Institute for Health Research (NIHR) School for Public Health Research (SPHR), Grant Reference Number PD-SPH-2015. The NIHR School for Public Health Research is a partnership between the Universities of: Sheffield, Bristol, Cambridge, Imperial, and University College London, The London School of Hygiene and Tropical Medicine, LiLaC—a collaboration between the Universities of Liverpool and Lancaster, and Fuse—the Centre for Translational Research in Public Health, a collaboration between Newcastle, Durham, Northumbria, Sunderland, and Teesside Universities. The views expressed are those of the author(s) and not necessarily those of the NIHR or the Department of Health and Social Care. SS was also supported by NIHR Northwest London Applied Research Collaboration (NW London ARC) and holds grants from The NIHR Imperial Biomedical Research Centre and The Daily Mile Foundation. KH was funded by the Wellcome Trust (107337/Z/15/Z).

## Author Disclaimer

The views expressed are those of the author(s) and not necessarily those of the NIHR or the Department of Health and Social Care.

## Conflict of Interest

HB has received speaker fees for talks on infant sleep from La Leche League Hungary, Laktation Berlin (Breastfeeding conference), La Leche League GB and GOLD conference (Lactiation conference), outside of the submitted work. The remaining authors declare that the research was conducted in the absence of any commercial or financial relationships that could be construed as a potential conflict of interest.

## Publisher's Note

All claims expressed in this article are solely those of the authors and do not necessarily represent those of their affiliated organizations, or those of the publisher, the editors and the reviewers. Any product that may be evaluated in this article, or claim that may be made by its manufacturer, is not guaranteed or endorsed by the publisher.

## Abbreviations

BCSQ: Brief Child Sleep Questionnaire
CCTQ: Children's ChronoType Questionnaire
COSMIN: COnsensus-based Standards for the selection of health Measurement Instruments
CSHQ: Children's Sleep Habits Questionnaire
SSTQ: Sleep Schedule Time Questionnaire
WHO: World Health Organization.
