# Supplementary material for: The Validity, Reliability, and Feasibility of Measurement Tools Used to Assess Sleep of Pre-school Aged Children: A Systematic Rapid Review
Source: Front Pediatr. 2021 Nov 26;9:770262. doi: 10.3389/fped.2021.770262 (PMC8662360; doi:10.3389/fped.2021.770262)
Supplement: Supplementary file 1 [file Data_Sheet_1.docx]

**Supplementary file 1: PRISMA Checklist**

| **Section and Topic** | **Item #** | **Checklist item** | **Location where item is reported** |
| --- | --- | --- | --- |
| **TITLE** | | |  |
| Title | 1 | Identify the report as a systematic review. | Page 1 |
| **ABSTRACT** | | |  |
| Abstract | 2 | See the PRISMA 2020 for Abstracts checklist. | Page 2 |
| **INTRODUCTION** | | |  |
| Rationale | 3 | Describe the rationale for the review in the context of existing knowledge. | Pages 3-4 |
| Objectives | 4 | Provide an explicit statement of the objective(s) or question(s) the review addresses. | Page 4 |
| **METHODS** | | |  |
| Eligibility criteria | 5 | Specify the inclusion and exclusion criteria for the review and how studies were grouped for the syntheses. | Pages 5-6 |
| Information sources | 6 | Specify all databases, registers, websites, organisations, reference lists and other sources searched or consulted to identify studies. Specify the date when each source was last searched or consulted. | Page 6, and supplementary file 2 |
| Search strategy | 7 | Present the full search strategies for all databases, registers and websites, including any filters and limits used. | Supplementary file 2 |
| Selection process | 8 | Specify the methods used to decide whether a study met the inclusion criteria of the review, including how many reviewers screened each record and each report retrieved, whether they worked independently, and if applicable, details of automation tools used in the process. | Page 5 |
| Data collection process | 9 | Specify the methods used to collect data from reports, including how many reviewers collected data from each report, whether they worked independently, any processes for obtaining or confirming data from study investigators, and if applicable, details of automation tools used in the process. | Page 6 |
| Data items | 10a | List and define all outcomes for which data were sought. Specify whether all results that were compatible with each outcome domain in each study were sought (e.g. for all measures, time points, analyses), and if not, the methods used to decide which results to collect. | Page 6 |
|  | 10b | List and define all other variables for which data were sought (e.g. participant and intervention characteristics, funding sources). Describe any assumptions made about any missing or unclear information. | Page 6 |
| Study risk of bias assessment | 11 | Specify the methods used to assess risk of bias in the included studies, including details of the tool(s) used, how many reviewers assessed each study and whether they worked independently, and if applicable, details of automation tools used in the process. | Page 6 |
| Effect measures | 12 | Specify for each outcome the effect measure(s) (e.g. risk ratio, mean difference) used in the synthesis or presentation of results. | Table 2 |
| Synthesis methods | 13a | Describe the processes used to decide which studies were eligible for each synthesis (e.g. tabulating the study intervention characteristics and comparing against the planned groups for each synthesis (item #5)). | Table 1, supplementary files 5-9 |
|  | 13b | Describe any methods required to prepare the data for presentation or synthesis, such as handling of missing summary statistics, or data conversions. | Page 6 and Table 2 |
|  | 13c | Describe any methods used to tabulate or visually display results of individual studies and syntheses. | Table 4, and supplementary files 5-9 |
|  | 13d | Describe any methods used to synthesize results and provide a rationale for the choice(s). If meta-analysis was performed, describe the model(s), method(s) to identify the presence and extent of statistical heterogeneity, and software package(s) used. | Page 6 and 8 |
|  | 13e | Describe any methods used to explore possible causes of heterogeneity among study results (e.g. subgroup analysis, meta-regression). | N/A |
|  | 13f | Describe any sensitivity analyses conducted to assess robustness of the synthesized results. | N/A |
| Reporting bias assessment | 14 | Describe any methods used to assess risk of bias due to missing results in a synthesis (arising from reporting biases). | Pages 6-8 |
| Certainty assessment | 15 | Describe any methods used to assess certainty (or confidence) in the body of evidence for an outcome. | Pages 6-8 |
| **RESULTS** | | |  |
| Study selection | 16a | Describe the results of the search and selection process, from the number of records identified in the search to the number of studies included in the review, ideally using a flow diagram. | Page 7 and Figure 1 |
|  | 16b | Cite studies that might appear to meet the inclusion criteria, but which were excluded, and explain why they were excluded. | Supplementary file 3 |
| Study characteristics | 17 | Cite each included study and present its characteristics. | Pages 7 and supplementary files 5-9 |
| Risk of bias in studies | 18 | Present assessments of risk of bias for each included study. | Pages 7-8 and supplementary file 4 |
| Results of individual studies | 19 | For all outcomes, present, for each study: (a) summary statistics for each group (where appropriate) and (b) an effect estimate and its precision (e.g. confidence/credible interval), ideally using structured tables or plots. | Supplementary files 5-9 |
| Results of syntheses | 20a | For each synthesis, briefly summarise the characteristics and risk of bias among contributing studies. | Pages 7-10 |
|  | 20b | Present results of all statistical syntheses conducted. If meta-analysis was done, present for each the summary estimate and its precision (e.g. confidence/credible interval) and measures of statistical heterogeneity. If comparing groups, describe the direction of the effect. | N/A |
|  | 20c | Present results of all investigations of possible causes of heterogeneity among study results. | N/A |
|  | 20d | Present results of all sensitivity analyses conducted to assess the robustness of the synthesized results. | N/A |
| Reporting biases | 21 | Present assessments of risk of bias due to missing results (arising from reporting biases) for each synthesis assessed. | Pages 7-8, and included in Table 4. |
| Certainty of evidence | 22 | Present assessments of certainty (or confidence) in the body of evidence for each outcome assessed. | Pages 7-8, and included in Table 4. Also included throughout results and discussion. |
| **DISCUSSION** | | |  |
| Discussion | 23a | Provide a general interpretation of the results in the context of other evidence. | Pages 11-13 |
|  | 23b | Discuss any limitations of the evidence included in the review. | Pages 11-13 |
|  | 23c | Discuss any limitations of the review processes used. | Page 13 |
|  | 23d | Discuss implications of the results for practice, policy, and future research. | Page 13 |
| **OTHER INFORMATION** | | |  |
| Registration and protocol | 24a | Provide registration information for the review, including register name and registration number, or state that the review was not registered. | Page 5 |
|  | 24b | Indicate where the review protocol can be accessed, or state that a protocol was not prepared. | Page 5 |
|  | 24c | Describe and explain any amendments to information provided at registration or in the protocol. | N/A |
| Support | 25 | Describe sources of financial or non-financial support for the review, and the role of the funders or sponsors in the review. | Page 14 |
| Competing interests | 26 | Declare any competing interests of review authors. | Page 14 |
| Availability of data, code and other materials | 27 | Report which of the following are publicly available and where they can be found: template data collection forms; data extracted from included studies; data used for all analyses; analytic code; any other materials used in the review. | Data used for all analyses can be found in supplementary files 5-9 |

*From:*  Page MJ, McKenzie JE, Bossuyt PM, Boutron I, Hoffmann TC, Mulrow CD, et al. The PRISMA 2020 statement: an updated guideline for reporting systematic reviews. BMJ 2021;372:n71. doi: 10.1136/bmj.n71

For more information, visit: <http://www.prisma-statement.org/>

**Supplementary File 2: Full Search Strategy**

Initial searches conducted on PROSPERO, electronic databases (Scopus, Web of Science, PsycARTICLES, PsycINFO, MEDLINE, CINAHL and SPORTDiscus), AND topic specific journals to identify pre-existing reviews. Citation searches of relevant reviews to identify further reviews.

| Source of Search Conducted | Date of Search | Searches Conducted | Search Outcome (n) |
| --- | --- | --- | --- |
| Web of Science (*Web of Science)* | 14/04/21 | ALL=(sleep) AND ALL= (valid* OR reliab* OR feasib*) AND ALL= (pre$school* OR "early years" OR "early childhood" OR "young children" OR kindergarten*) AND ALL= (assess* OR measur* OR method*) | 464 |
| Medline (*Ovid*) | 14/04/21 | (sleep and (valid* or reliab* or feasib*) and (pre$school* or "early years" or "early childhood" or "young children" or kindergarten*) and (assess* or measur* or method*)).af. | 788 |
| APA PsycARTICLES, APA PsycINFO, SPORTdiscus (*EBSCOhost)* | 14/04/21 | TX SLEEP AND TX ( assess* OR measur* OR method*) AND TX ( valid* OR reliab* OR feasib* ) AND TX ( pre#school* OR "early childhood" OR "early years" OR "young children" OR kindergarten* ) | 2425 |
| Scopus (*Science Direct*) | 14/04/21 | ( TITLE-ABS-KEY ( "sleep" )  AND  TITLE-ABS-KEY ( ( pre*school*  OR  "early years"  OR  "early childhood"  OR  "young children"  OR  kindergarten* ) )  AND  TITLE-ABS-KEY ( ( assess*  OR  measure*  OR  method* ) )  AND  TITLE-ABS-KEY ( ( valid*  OR  reliab*  OR  feasib* ) ) ) | 976 |
| Trial registries- International Clinical Trials Registry Platform | 14/04/21 | SLEEP AND CHILD* | 703 |
| Grey literature- opengrey.eu | 14/04/21 | sleep AND "preschool*" OR "early years" OR "early childhood" OR "young children" OR "kindergarten*" | 1068 |
| Sleep Health | 04/05/21 | pre*school* OR "early years" OR "early childhood" OR "young children" OR kindergarten* | 153 |
| Sleep | 04/05/21 | "preschool" OR "pre-school" OR "early years" OR "early childhood" OR "young children" OR "kindergarten" | 746 |
| Reference lists of existing reviews |  | Reference lists | 1029 |
| Citation searching/reference list searching of included studies | During data extraction | Reference lists/citation searching |  |

**Supplementary file 3: Excluded studies with reasons**

**Part 1: Databases**

**Children outside of age range (n=38)**

**Children <3 years old (n=8)**

1. Belanger ME, Bernier A, Paquet J, Simard V, Carrier J. Validating Actigraphy as a Measure of Sleep for Preschool Children. Journal of Clinical Sleep Medicine. 2013;9(7):701-6.

2. Eaton CK, Henning E, Lam J, Paasch V. Actigraphy technology: Informing assessment and intervention for sleep disturbances in young children. Clinical Practice in Pediatric Psychology. 2019;7(4):347-57.

3. Goodlin-Jones BL, Sitnick SL, Tang K, Liu J, Anders TF. The Children's Sleep Habits Questionnaire in toddlers and preschool children. Journal of Developmental & Behavioral Pediatrics.29(2):82-8.

4. Hatch B, Nordahl CW, Schwichtenberg AJ, Ozonoff S, Miller M. Factor Structure of the Children's Sleep Habits Questionnaire in Young Children with and Without Autism. Journal of Autism and Developmental Disorders.12.

5. Henderson JA, Jordan SS. Development and preliminary evaluation of the bedtime routines questionnaire. Journal of Psychopathology and Behavioral Assessment. 2010;32(2):271-80.

6. Minde K, Popiel K, Leos N, Falkner S, Parker K, Handleyderry M. THE EVALUATION AND TREATMENT OF SLEEP DISTURBANCES IN YOUNG-CHILDREN. Journal of Child Psychology and Psychiatry. 1993;34(4):521-33.

7. Sneddon P, Peacock GG, Crowley SL. Assessment of sleep problems in preschool aged children: an adaptation of the children's sleep habits questionnaire. Behavioral Sleep Medicine.11(4):283-96.

8. Steur LMH, Visser EH, Grootenhuis MA, Terwee CB, Kaspers GJL, van Litsenburg RRL. Psychometric properties and Dutch norm values of the Children's Sleep Habits Questionnaire in toddlers. Sleep Medicine.34:57-63.

**Children >7 years old (n=20)**

1. Bevans KB, Meltzer LJ, De La Motte A, Kratchman A, Viel D, Forrest CB. Qualitative Development and Content Validation of the PROMIS Pediatric Sleep Health Items. Behavioral Sleep Medicine.17(5):657-71.

2. Blanc F, Merklen F, Blanchet C, Mondain M, Akkari M. Respiratory polygraphy in children: Feasibility in everyday practice in an ENT department and value of automatic detection of respiratory events. European annals of otorhinolaryngology, head & neck diseases.136(4):235-40.

3. Certal V, de Lima FF, Winck JC, Azevedo I, Costa-Pereira A. Translation and cross-cultural adaptation of the Pediatric Sleep Questionnaire into Portuguese language. International Journal of Pediatric Otorhinolaryngology.79(2):175-8.

4. Dollinger SJ, Schum RL. The Missouri Children's Picture Series: Utility of the sleep disturbance scale with a clinical sample. Journal of Pediatric Psychology. 1979;4(2):113-8.

5. Forrest CB, Meltzer LJ, Marcus CL, De La Motte A, Kratchman A, Buysse DJ, et al. Development and validation of the PROMIS Pediatric Sleep Disturbance and Sleep-Related Impairment item banks. Sleep. 2018;41(6).

6. Goodwin JL, Enright PL, Kaemingk KL, Rosen GM, Morgan WJ, Fregosi RF, et al. Feasibility of using unattended polysomnography in children for research--report of the Tucson Children's Assessment of Sleep Apnea study (TuCASA). Sleep.24(8):937-44.

7. Hyde M, O'Driscoll DM, Binette S, Galang C, Tan SK, Verginis N, et al. Validation of actigraphy for determining sleep and wake in children with sleep disordered breathing. Journal of Sleep Research. 2007;16(2):213-6.

8. Ioan I, Weick D, Schweitzer C, Guyon A, Coutier L, Franco P. Feasibility of parent-attended ambulatory polysomnography in children with suspected obstructive sleep apnea. Journal of Clinical Sleep Medicine. 2020;16(7):1013-9.

9. Iwasaki M, Iemura A, Oyama T, Matsuishi T. A Novel Subjective Sleep Assessment Tool for Healthy Elementary School Children in Japan. Journal of Epidemiology. 2010;20:S476-S81.

10. Kuwada A, Mohri I, Asano R, Matsuzawa S, Kato-Nishimura K, Hirata I, et al. Japanese Sleep Questionnaire for Elementary Schoolers (JSQ-ES): validation and population-based score distribution. Sleep Medicine. 2018;41:69-77.

11. Marcus CL, Traylor J, Biggs SN, Roberts RS, Nixon GM, Narang I, et al. Feasibility of comprehensive, unattended ambulatory polysomnography in school-aged children. Journal of Clinical Sleep Medicine.10(8):913-8.

12. Owens JA, Spirito A, McGuinn M. The Children's Sleep Habits Questionnaire (CSHQ): psychometric properties of a survey instrument for school-aged children. Sleep.23(8):1043-51.

13. Perdahli Fiş N, Arman A, Ay P, Topuzoğlu A, Güler AS, Gökçe Imren S, et al. Çocuk uyku alışkanlıkları anketinin Türkçe geçerliliği ve güvenilirliği = The validity and the reliability of Turkish Version of Children’s Sleep Habits Questionnaire. Anadolu Psikiyatri Dergisi. 2010;11(2):151-60.

14. Schlarb AA, Schwerdtle B, Hautzinger M. Validation and psychometric properties of the German version of the Children's Sleep Habits Questionnaire (CSHQ-DE). Somnologie. 2010;14(4):260-6.

15. Smith C, Galland B, Taylor R, Meredith-Jones K. ActiGraph GT3X+ and Actical Wrist and Hip Worn Accelerometers for Sleep and Wake Indices in Young Children Using an Automated Algorithm: Validation With Polysomnography. Frontiers in Psychiatry. 2020;10.

16. Spruyt K, Gozal D, Dayyat E, Roman A, Molfese D. Sleep assessments in healthy school‐aged children using actigraphy: Concordance with polysomnography. Journal of Sleep Research. 2011;20(1):223-32.

17. Tamura T, Togawa T, Murata M. A bed temperature monitoring system for assessing body movement during sleep. Clinical Physics & Physiological Measurement.9(2):139-45.

18. Waumans RC, Terwee CB, Van den Berg G, Knol DL, Van Litsenburg RR, Gemke RJ. Sleep and sleep disturbance in children: Reliability and validity of the Dutch version of the Child Sleep Habits Questionnaire. Sleep.33(6):841-5.

19. Werner H, LeBourgeois MK, Geiger A, Jenni OG. Assessment of chronotype in four- to eleven-year-old children: Reliability and validity of the Children's ChronoType Questionnaire (CCTQ). Chronobiology International. 2009;26(5):992-1014.

20. Yosaee S, Gharamaleki AS, Zamani A, Khosravi A, Djafarian K. Validation of self-reported sleep against actigraphy. Journal of Zanjan University of Medical Sciences and Health Services. 2013;21(88).

**Children <3 years and >7 years (n=8)**

1. Bastida‐Pozuelo MF, Sánchez‐Ortuño MM. Preliminary analysis of the concurrent validity of the Spanish translation of the BEARS sleep screening tool for children. Journal of Psychiatric and Mental Health Nursing. 2016;23(8):513-20.

2. Bonuck KA, Goodlin-Jones BL, Schechter C, Owens J. Modified Children's sleep habits questionnaire for behavioral sleep problems: A validation study. Sleep Health. 2017;3(3):136-41.

3. Lebourgeois MK. Validation of the Children's Sleep-Wake Scale: ProQuest Information & Learning; 2003.

4. LeBourgeois MK, Harsh JR. Development and psychometric evaluation of the Children's Sleep-Wake Scale. Sleep Health.2(3):198-204.

5. McGreavey JA, Donnan PT, Pagliari HC, Sullivan FM. The Tayside children's sleep questionnaire: A simple tool to evaluate sleep problems in young children. Child: Care, Health and Development. 2005;31(5):539-44.

6. Rosen D. Many parents report their childs breathing and sleep patterns during overnight sleep study as atypical. Clinical Pediatrics. 2010;49(8):764-7.

7. Shirouzu S, Seno Y, Tobioka K, Yagi T, Takahashi T, Sasaki M, et al. For Children's Sleep Assessment: Can we trace the change of sleep depth based on ECG data measured at their respective home with a wearable device? 2016 2016. 208-11 p.

8. Wu XM, Bennett DH, Lee K, Cassady DL, Ritz B, Hertz-Picciotto I. Feasibility of using web surveys to collect time-activity data. Journal of Exposure Science and Environmental Epidemiology. 2012;22(2):116-25.

**Children outside age range / sleep disordered breathing- unable to distinguish the pre-school aged children without SDB (n=2)**

1. Meltzer LJ, Walsh CM, Traylor J, Westin AM. Direct comparison of two new actigraphs and polysomnography in children and adolescents. Sleep.35(1):159-66.
2. Meltzer LJ, Walsh CM, Peightal AA. Comparison of actigraphy immobility rules with polysomnographic sleep onset latency in children and adolescents. Sleep and Breathing. 2015;19(4):1415-23.

**Children with disability/limiting illness (n=1)**

1. Lesser AD, Luczynski KC, Hood SA. Evaluating motion detection to score sleep disturbance for children: A translational approach to developing a measurement system. Journal of Applied Behavior Analysis. 2019;52(2):580-99.

**Purpose to determine sleep disturbances/diagnoses (n=4)**

1. Poels PJP, Schilder AGM, Van Den Berg S, Hoes AW, Joosten KFM. Evaluation of a New Device for Home Cardiorespiratory Recording in Children. Archives of Otolaryngology - Head and Neck Surgery. 2003;129(12):1281-4.
2. Romeo DM, Bruni O, Brogna C, Ferri R, Galluccio C, De Clemente V, et al. Application of the sleep disturbance scale for children (SDSC) in preschool age. European Journal of Paediatric Neurology.17(4):374-82.
3. Shimizu S, Kato-Nishimura K, Mohri I, Kagitani-Shimono K, Tachibana M, Ohno Y, et al. Psychometric properties and population-based score distributions of the Japanese Sleep Questionnaire for Preschoolers. Sleep Medicine.15(4):451-8.
4. Gill AI, Schaughency E, Gray A, Galland BC. Reliability of home-based physiological sleep measurements in snoring and non-snoring 3-year olds. Sleep & Breathing.17(1):147-56.

**Did not explore utility of tool (n=3)**

1. Jafar NK, Tham EKH, Eng DZH, Goh DYT, Teoh OH, Lee YS, et al. The association between chronotype and sleep problems in preschool children. Sleep Medicine. 2017;30:240-4.

2. Lo MJ. Relationship between sleep habits and nighttime sleep among healthy preschool children in taiwan. Annals of the Academy of Medicine Singapore. 2016;45(12):549-56.

3. Tey C, Wake M, Campbell M, Hampton A, Williams J. The Light Time-Use Diary and preschool activity patterns: exploratory study. International Journal of Pediatric Obesity.2(3):167-73.

**Normative sleep data parameters/ sleep wake patterns (n=3)**

1. Scholle S, Beyer U, Bernhard M, Eichholz S, Erler T, Graness P, et al. Normative values of polysomnographic parameters in childhood and adolescence: quantitative sleep parameters. Sleep Medicine.12(6):542-9.
2. Scholle S, Wiater A, Scholle HC. Normative values of polysomnographic parameters in childhood and adolescence: cardiorespiratory parameters. Sleep Medicine.12(10):988-96.
3. Acebo C, Sadeh A, Seifer R, Tzischinsky O, Hafer A, Carskadon MA. Sleep/wake patterns derived from activity monitoring and maternal report for healthy 1-to 5-year-old children. Sleep. 2005;28(12):1568-77.

**Not measuring sleep (n=1)**

1. Chuang SY, Teng A, Butler JE, Gandevia SC, Selvadurai H, Jaffe A. Validation of a quantitative method to measure neural respiratory drive in children during sleep. Respiratory Physiology & Neurobiology.239:75-80.

**Parent/child interactions rather than sleep duration/quality (n=3)**

1. Alfano CA, Smith VC, Reynolds KC, Reddy R, Dougherty LR. The Parent-Child Sleep Interactions Scale (PSIS) for Preschoolers: Factor Structure and Initial Psychometric Properties. Journal of Clinical Sleep Medicine. 2013;9(11):1153-60.

2. Barrios CS, Jay SY, Smith VC, Alfano CA, Dougherty LR. Stability and Predictive Validity of the Parent-Child Sleep Interactions Scale: A Longitudinal Study Among Preschoolers. Journal of Clinical Child & Adolescent Psychology.47(3):382-96.

3. Smith VC, Leppert KA, Alfano CA, Dougherty LR. Construct validity of the parent-child sleep interactions scale (PSIS): associations with parenting, family stress, and maternal and child psychopathology. Sleep Medicine.15(8):942-51.

**Number of days monitoring – measuring stability of the behaviour rather than measurement properties of the tool (n=2)**

1. Acebo C, Sadeh A, Seifer R, Tzischinsky O, Wolfson AR, Hafer A, et al. Estimating sleep patterns with activity monitoring in children and adolescents: how many nights are necessary for reliable measures? Sleep.22(1):95-103.

2. Taylor RW, Williams SM, Farmer VL, Taylor BJ. The stability of sleep patterns in children 3 to 7 years of age. Journal of Pediatrics.166(3):697-702.e1.

**Abstract with insufficient information available (n=5)**

1. Appleyard K, Galland B, Gill A, Lobb C, Schaughency E. An objective assessment of the reliability of parental reports of snoring in preschool children. Journal of Sleep Research. 2011;20:17-8.

2. DiLeo HA, Umlauf MG, Makris CM, Orji C. Assessment of the reliability and validity of one brand of actigraphy device in young children. Sleep. 2005;28:A318-A.

3. Gomes AM, Figueiredo S, Rocha C, Ferreira E, Silva B, Caldas AC, et al. SLEEP QUALITY AND CHRONOTYPE OF PORTUGUESE SCHOOL-AGED CHILDREN. 2017 2017. 1-16 p.

4. Mellecker RR, McManus AM, Matsuzaka A. Validity and Reliability of the Sedentary Behavior and Sleep Scale (SBSS) in Young Hong Kong Chinese Children. Asian Journal of Exercise & Sports Science. 2012;9(1):21-36.

5. Randler C, Truc Y. Adaptation of the Composite Scale of Morningness for parent report and results from kindergarten children. Swiss Journal of Psychology. 2014;73(1):35-9.

**Part 2: Trial registries**

**Children <3 years and >7 years (n=1)**

[ICTRP Search Portal (ictrptest.azurewebsites.net)](https://ictrptest.azurewebsites.net/Trial2.aspx?TrialID=NCT00928993)

**Part 3: Citation searches/reference lists of included studies**

**Children outside age range**

**Children > 7 years (n=9)**

1. Berger, I., Obeid, J., Timmons, B. W., & DeMatteo, C. (2017). Exploring accelerometer versus self-report sleep assessment in youth with concussion. Global pediatric health, 4, 2333794X17745973
2. Colley, R. C., Wong, S. L., Garriguet, D., Janssen, I., Gorber, S. C., & Tremblay, M. S. (2012). Physical activity, sedentary behaviour and sleep in Canadian children: parent-report versus direct measures and relative associations with health risk. Health reports, 23(2), A1.;
3. Dayyat E, Spruyt K, Molfese D, Gozal D. Sleep estimates in children: parental versus actigraphic assessments. Nat Sci Sleep. (2011) 3:115–23. 10.2147/NSS.S25676;
4. Goodwin, J. L., Silva, G. E., Kaemingk, K. L., Sherrill, D. L., Morgan, W. J., & Quan, S. F. (2007). Comparison between reported and recorded total sleep time and sleep latency in 6-to 11-year-old children: the Tucson Children’s Assessment of Sleep Apnea Study (TuCASA). Sleep and Breathing, 11(2), 85-92;
5. Holley, S., Hill, C. M., & Stevenson, J. (2010). A comparison of actigraphy and parental report of sleep habits in typically developing children aged 6 to 11 years. Behavioral sleep medicine, 8(1), 16-27.
6. Lockley SW, Skene DJ, Arendt J. Comparison between subjective and actigraphic measurement of sleep and sleep rhythms. Journal of Sleep Research. 1999;9:175–183;
7. Loureiro, H. C., Pinto, T. R., Pinto, J. C., Pinto, H. R., & Paiva, T. (2013). Validation of the children sleep habits questionnaire and the sleep self report for Portuguese children. Sleep Science, 6(4), 151-158
8. Markovich, A. N., Gendron, M. A., & Corkum, P. V. (2015). Validating the Children’s Sleep Habits Questionnaire against polysomnography and actigraphy in school-aged children. Frontiers in psychiatry, 5, 188.
9. Mazza, S., Bastuji, H., & Rey, A. E. (2020). Objective and Subjective Assessments of Sleep in Children: Comparison of Actigraphy, Sleep Diary Completed by Children and Parents’ Estimation. Frontiers in Psychiatry, 11.

**Children <3 years (n=4)**

1. Bélanger, M. È., Bernier, A., Paquet, J., Simard, V., & Carrier, J. (2013). Validating actigraphy as a measure of sleep for preschool children. Journal of Clinical Sleep Medicine, 9(7), 701-706
2. Covington, L. B., Rogers, V. E., & Black, M. M. (2019). Sleep Measurement in Toddlers From Low-income Families. Journal of Developmental & Behavioral Pediatrics, 40(3), 219-223.
3. Liu Z, Wang G, Tang H, Wen F, Li N. Reliability and validity of the Children's sleep habits questionnaire in preschool-aged Chinese children. Sleep Biol Rhythms. 2014; 12(3):187–193
4. Ward T, Gay C, Anders TF, Alkon A, Lee KA. Sleep and napping patterns in 3- to 5-year-old children attending full-day childcare centers. Journal of Pediatric Psychology. 2007;33:666–672

**Children with clinically diagnosed disturbances/disorders (n=3)**

1. Sadeh, A., Lavie, P., Scher, A., Tirosh, E., & Epstein, R. (1991). Actigraphic home-monitoring sleep-disturbed and control infants and young children: a new method for pediatric assessment of sleep-wake patterns. Pediatrics, 87(4), 494-499.
2. Toon, E., Davey, M. J., Hollis, S. L., Nixon, G. M., Horne, R. S., & Biggs, S. N. (2016). Comparison of commercial wrist-based and smartphone accelerometers, actigraphy, and PSG in a clinical cohort of children and adolescents. *Journal of Clinical Sleep Medicine*, *12*(3), 343-350.
3. Osterbauer, B., Koempel, J. A., Ward, S. L. D., Fisher, L. M., & Don, D. M. (2016). A comparison study of the Fitbit activity monitor and PSG for assessing sleep patterns and movement in children. *Journal of Otolaryngology Advances*, *1*(3), 24.

**Did not explore utility of tool (n=6)**

1. Belísio, A. S., Kolodiuk, F. F., Louzada, F. M., Valdez, P., & Azevedo, C. V. (2017). Sleep–Wake Cycle, Daytime Sleepiness, and Attention Components in Children Attending Preschool in the Morning and Afternoon Shifts. Mind, Brain, and Education, 11(1), 10-20.
2. Iwata, S., Iwata, O., Iemura, A., Iwasaki, M., & Matsuishi, T. (2012). Sleep architecture in healthy 5‐year‐old preschool children: associations between sleep schedule and quality variables. Acta Paediatrica, 101(3), e110-e114.;
3. Sadeh, A. (2008). Commentary: Comparing actigraphy and parental report as measures of children's sleep. Journal of pediatric psychology, 33(4), 406-407
4. Silva E, Simões P, Macedo M, Duarte J, Silva D. Parents' perception of the sleep habits and quality of preschool-aged children. Rev Enfermagem Ref. (2018) IV:63–70. 10.12707/RIV17103;
5. Tininenko, J. R. (2008). Actigraphic evaluation of sleep disturbance in young children (Doctoral dissertation, University of Oregon).
6. Touchette, E., Mongrain, V., Petit, D., Tremblay, R. E., & Montplaisir, J. Y. (2008). Development of sleep-wake schedules during childhood and relationship with sleep duration. Archives of pediatrics & adolescent medicine, 162(4), 343-349

**Insufficient information available (n=2)**

1. Lotjonen, J. M. (2001). Sleep/wake detection using an active security device. *Sleep*, *24*, S399.
2. Doi, Y., Ishihara, K., Uchiyama M., Takimoto, H. (2013). Development of the Japanese Version of Children’s Chronotype Questionnaire. Japan Journal Sleep Medicine. 7:486-93 (In Japanese).

**Part 4: Journal articles**

**Did not explore utility of tool (n=1)**

1. Lionetti, F., Dellagiulia, A., Verderame, C., Sperati, A., Bodale, G., Spinelli, M., & Fasolo, M. (2021). The Children's Sleep Habits Questionnaire: identification of sleep dimensions, normative values, and associations with behavioral problems in Italian preschoolers. *Sleep Health*.

**Children <3 years (n=1)**

1. Olivia Cicalese, Ariel Williamson, Jodi Mindell, Lisa Meltzer, 592 Objective vs. Subjective Sleep Data in Early Childhood: Implications for Health Disparities Research, Sleep, Volume 44, Issue Supplement_2, May 2021, Page A233, <https://doi.org/10.1093/sleep/zsab072.590>

**Supplementary file 4: Risk of bias of included studies**


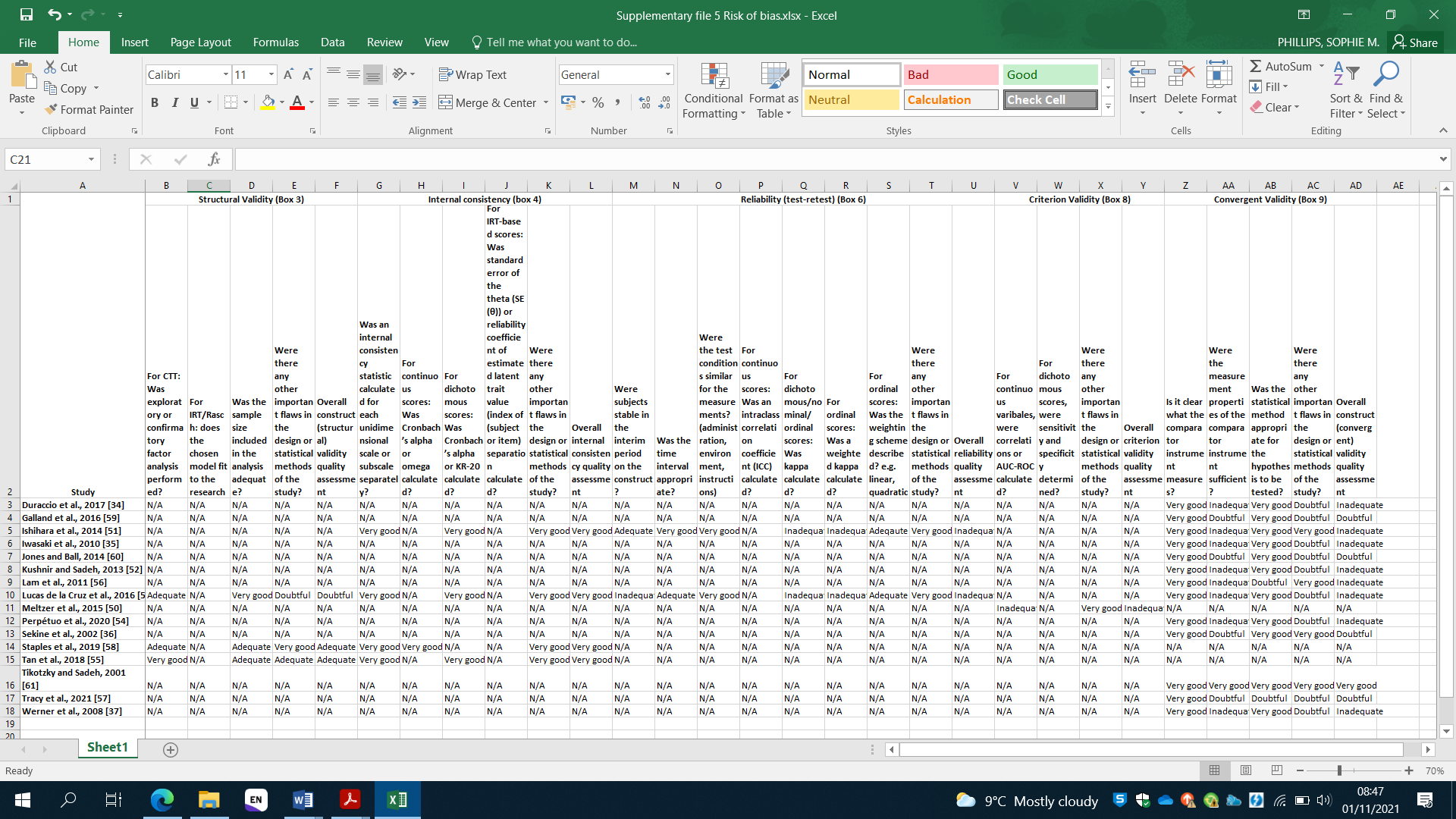


**Supplementary file 5: Construct (structural) validity of measurement tools used to assess sleep of pre-school aged children**

| **Study** | **Study sample** | **Measurement tool under study** | **Methods** | **Structural Validity Results** | **Risk of bias** |
| --- | --- | --- | --- | --- | --- |
| Lucas-de la Cruz et al., 2016 [53] | Spain;  N=286;  4-7 years; | Child Sleep Habits Questionnaire (CSHQ)- Spanish version | Parents completed the Child Sleep Habits Questionnaire (CSHQ)- Spanish version | **Exploratory factor analysis conducted** – 10 factors with eigenvalues >1. When analysed items’ content in each subscale they did not have logical connection. Eight factor model could be appropriated but many items not allocated in correct subscales.  Kept the same factor structure as original questionnaire. | Doubtful |
| Tan et al., 2018 [55] | N=171;  4-5 years; 53% female, 47% male;  China  Chinese kindergarteners.  Most parents worked full-time. Over 80% of mothers and 85% fathers had at least a college level education. Over half of the mothers and 80% fathers made ¥5000 or more monthly. | Children’s Sleep Habits Questionnaire (CSHQ)- Chinese version | Parents completed the Chinese version of the CSHQ-C | **Confirmatory factor analysis –**Concluded that none of the existing factor structures (5 factor, 8 factor, 4 factor) outlined in previous studies and the original questionnaire were suitable for the data with this age group.  **Exploratory factor analysis** – 6 factor structure best comparative fit = 0.89, followed this up with item reduction and confirmatory factor analysis with 4 factor structure: bedtime behaviours (**α** =.59), sleep behaviours (α=0.62), morning wakings (α=0.69) and daytime sleepiness (α=0.67). | Adequate |
| Staples et al., 2019 [58] | N=107 (at 3 years), n=104 (at 3.5 years);  3-3.5 years;  Sex not reported for this age category;  USA  Information on parental education, ethnicity and socioeconomic status was available but for the whole sample, not separated for age groups of interest. Predominantly college educated parents, European American, high socioeconomic status. | MicroMini Motionlogger Accelerometer;  *Wrist worn;*  *Scored using Action W-2 software and Sadeh algorithm^63^*  *Sleep diary also completed to record bedtime, night awakenings, rise times and other issues relating to child’s sleep.* | Accelerometer worn for 7 days at three time points in longitudinal study (2.5 years, 3 years, 3.5 years). | **Principal component analysis (with oblique rotation):**  Strong loadings for a four factor structure at both time points, explaining 82% of the variance (when child aged 3) and 81% (when child age 3.5 years):   1. **Sleep activity**   -Average time awake after sleep onset  -Average minute to minute activity level  -Average n of night awakenings lasting 5 minutes or more  -Average duration of longest wake episode after sleep onset  -Average % of active epochs after sleep onset   1. **Sleep variability**   -Time of sleep onset  -Duration of time in bed  -Duration of sleep period  -Time of midsleep  -Bedtime  -Minutes asleep whilst in bed   1. **Sleep timing**   -Average time of midsleep  -Average time of sleep onset  -Average bedtime   1. **Sleep duration**   -Average sleep period  -Average duration of time in bed  -Average minutes asleep while in bed  The daytime sleep variables (duration and variability) and night time latency to sleep onset were indicated to be independent from the 4 component structure when assessed with children aged 2.5 years, so were not included in these analyses. | Adequate |

**Supplementary file 6: Criterion validity of measurement tools used to assess sleep of pre-school aged children**

| **Study** | **Study sample** | **Measurement tool under study** | **Comparison measurement tool(s)** | **Methods** | **Outcome measures** | **Validity Results** | **Risk of bias** |
| --- | --- | --- | --- | --- | --- | --- | --- |
| Meltzer et al., 2015 [50] | N=14  3-5 years;  Sex not reported for this age category;  USA  Ethnicity reported for whole sample, but not separately by age category. | **Fitbit Ultra**  *Non-dominant wrist;*  *1 minute epoch;*  *Proprietary software algorithm for both normal and sensitive sleep recording modes.* | **Polysomnography (PSG):** Sandman 10.0 platform and **video and audio recordings.** | **Laboratory based (day of week unknown)**  Overnight polysomnography in the pediatric sleep laboratory at Children’s of Alabama. Children wore a Fitbit Ultra for the duration of the night. Some children randomly assigned to also wear two accelerometers, but results not available as to whether this included pre-school aged children. | **Both measurement tools:**   - Total sleep time - Wake after sleep onset - Sleep efficiency | Fitbit Ultra (Normal setting) significantly overestimated total sleep time and sleep efficiency (p<0.05). No significant difference for wake after sleep onset, but was lower from the Fitbit Ultra (26.6 minutes) than PSG (45.5 minutes).  Fitbit Ultra (sensitive mode) significantly underestimated total sleep time, overestimated wake after sleep onset and underestimated sleep efficiency (p<0.001). | Inadequate |

**Supplementary file 7: Convergent validity of measurement tools used to measure sleep of pre-school aged children**

***Accelerometers***

| **Study** | **Study sample** | **Measurement tool under study** | **Comparison measurement tool(s)** | **Methods** | **Outcome measures** | **Validity Results** | **Risk of bias rating** |
| --- | --- | --- | --- | --- | --- | --- | --- |
| Tracy et al. 2021 [57] | N=200;  3-6 years; 49% male, 51% female;  USA  Ethnicity and parental education reported for whole sample, but not separately for development and validation groups. However, overall sample was largely (90%) Hispanic/Latino. Majority of parents (62%) had less than a high school degree or equivalent. | **Actigraph GT3X+**  *Waist;*  *60 sec epoch;*  *Non wear periods identified and excluded using Choi’s algorithm ^79^* | Visual identification of bedrest and wake periods by two trained raters.  Parental reported sleep survey  Pre-existing algorithms: Sadeh’s automated sleep algorithm^63^and decision tree (DT) youth algorithm by Tracy et al., 2014 ^64^ | **Free living (weekday and weekend day required)**  Children wore the accelerometer on their waist for 7 days (including day time), a recording of ≥3 valid nights (of ≥6 hours) including ≥2 weeknights and ≥1 weeknight for inclusion. | **For all tools:**  Bedrest (sleep time) and wake time periods.  **DT algorithm and parental survey:**  Children taking naps, nap frequency and nap duration | **Independent samples t-test:**  DT performed better at identifying bedrest than DT-youth and Sadeh’s algorithm (p<0.001).  **Paired t-tests:**  Bedrest determined by DT was not significantly different from visual identification of the data (p=0.859), but was significantly shorter than Sadeh’s algorithm and parental survey (p<0.001).  Wake time determined by DT was not significantly longer from visual identification of the data (p=0.859), but was significantly different to Sadeh’s algorithm (p<0.001).  Bedrest determined by Sadeh’s algorithm was significantly different to parental survey and visual identification (p<0.001).  Wake time determined by Sadeh’s algorithm was significantly shorter than visual identification (p<0.001).  Bedrest determined by parental survey was significantly longer than from visual identification (p<0.001).  The number of children taking naps was significantly lower when determined by parental report than DT identified (p<0.001). Nap frequency was higher and duration longer when reported by parental report than DT identified (p<0.001).  **Overlap coefficients:**  Total sleep significantly different between parental report and DT, parental report and Sadeh’s algorithm and DT and Sadeh’s algorithm (all p<0.001).  (Inter-rater agreement for visual identification of data was very good *k=0.99)* | Doubtful |

***Questionnaires***

| **Study** | **Study sample** | **Measurement tool under study** | **Comparison measurement tool(s)** | **Methods** | **Outcome measures** | **Validity Results** | **Risk of bias rating** |
| --- | --- | --- | --- | --- | --- | --- | --- |
| Duraccio et al., 2017 [34] | N=108;  4-6 years;  42% female; 53% male; 5% missing  USA  86% Caucasian, 2% Hispanic, >1% Native American, 2% Asian, 5% Other, 5% Missing  Mean monthly income = $5,693.73  (Likely high socioeconomic profile as recruited through universities) | **Children’s Sleep Habits Questionnaire (CSHQ)**  *Parental reported typical week of sleep* | **Actigraph GT3X+**  *Right hip*  *15 sec epoch*  *Sadeh algorithm*^63^*, sleep time established by parental report in a demographics questionnaire of average bed and wake up time* | **Free living (week days only)**  CSHQ completed electronically, 3 days of continuous Actigraph GT3X+ wearing one week later | **CSHQ**:   - Sleep duration - Sleep onset delay - Night awakenings - Daytime sleepiness   **Actigraph**:   - Minutes asleep - Sleep latency - Minutes awake after sleep onset - Number of awakenings and average awakening length - Sleep efficiency | **Pearson’s Correlations:**  Minutes asleep (actigraph) and sleep duration (CSHQ) r= -0.13  Sleep latency (actigraph) and sleep onset delay (CHSQ) r=-0.16  Minutes awake after sleep onset (actigraph) and night awakenings (CHSQ) = r=-0.06,  N of awakenings (actigraph) and night awakenings (CSHQ) r=-0.05.  Average awakening length (actigraph) and night awakenings (CSHQ) r=0.16.  Sleep latency (Actigraph) and night awakenings (CSHQ)(r=0.30, p=0.002)  Sleep efficiency (actigraph) and daytime sleepiness (CSHQ) r=-0.01.  Actigraph minutes asleep were significantly correlated with CSHQ daytime sleepiness (r=-0.25, p=0.008).  Minutes asleep (Actigraph) and total score (CSHQ) (r=-0.25, p =0.009).  No significant differences for age or sex. | Inadequate |
| Ishihara et al., 2014 [51] | N=72;  3-6 years;  37 female, 35 male;  Japan | **Children's ChronoType Questionnaire (CCTQ)- Japanese version** | **Actiwatch 2 (Philips Respironics)**  *Non dominant wrist*  *60 sec epoch*  *Actiware software and* ***sleep diary*** *to determine sleep onset/offset and to check artifacts appearing on accelerometer recordings.* | **Free living (week day and weekend day- night time sleep only)**  Actiwatch worn for 7 days, concurrent parental reported sleep diary, and CCTQ completed at one time point during the week | **CCTQ and Actiwatch 2**:   - Bedtime - Time of lights off - Sleep onset time - Sleep offset time - Wake up time - Get up time - Midsleep point - Duration (time in bed, sleep period, sleep latency). | **Spearman’s Correlation:**  Sleep wake parameters measured with CCTQ were highly correlated to those reported or measured using Actiwatch (p<0.001). Correlation coefficients slightly smaller on free days than on scheduled days.  Sleep latency on scheduled days and free days, and sleep period on free days, the correlation coefficients were smaller than those of the remaining parameters.  Scheduled/weekday results: Bedtime r=0.774  Sleep onset time r=0.775  Wake up time/sleep offset time r=0.824  Get up time r=0.830  Midsleep point r=0.836  Time in bed r=0.740  Sleep period r=0.743  Sleep latency r=0.512.  Free day/weekend results:  Bedtime =0.641  Sleep onset time r=0.606  Wake up time/sleep offset time r=0.707  Get up time r= 0.714  Midsleep point r=0.786  Time in bed r=0.556  Sleep period r=0.490  Sleep latency r=0.380. | Inadequate |
| Iwasaki et al., 2010 [35]  *also examined a sleep diary | N=47;  5 years old;  21 female, 27 male;  Japan | **Brief questionnaire**  *12 major questions regarding child's sleep patterns and sleep quality based on past 4 weeks* | **Ambulatory Monitoring**  *Wrist (dominant or non-dominant)*  *60 sec epoch*  *Data analysed using Motion Logger ActFAST Analysis software, and sleep end time using Cole algorithm^69^* | **Free living (week day and weekend day- night time sleep only)**  Ambulatory monitoring and sleep diary completed daily for 7 days, brief questionnaire completed at the end of the study period | **Brief questionnaire:**   - Usual bedtime - Sleep onset time - Sleep end time - Sleep period - Number of night wakings (>5 minutes) - Sleep latency   **Ambulatory Monitoring:**   - Bedtime - Sleep onset time - Sleep latency - Sleep end time - Sleep period - Number of night wakings | **Pearson’s Correlations:**  **Brief questionnaire and ambulatory monitoring:**  ***Bedtime***  Overall r=0.17, p<0.01  Weekday r=0.25, p<0.01  Weekend r=0.02  ***Sleep onset time***  Overall r=0.39, p<0.001  Weekday r=0.49, p<0.01  Weekend r=0.07  ***Sleep latency***  Overall r=0.04  Weekday r=0.07  Weekend r=0.02  ***Sleep end time***  Overall r=0.51, p<0.001  Weekday r=0.59, p<0.001  Weekend r=0.08  **Sleep period**  Overall r=0.35, p<0.001  Weekday: r=0.38, p<0.001  Weekend r=0.01  **Night wakings**  Overall, weekday and weekend r=0.00  **Comparison between questionnaire and diary:**  ***Sleep onset time:***  Overall r=0.38, p<0.001  Weekday r=0.45, p<0.001  Weekend r=0.07  ***Sleep end time:***  Overall r=0.53, p<0.001  Weekday r=0.64, p<0.001  Weekend r=0.18, p<0.01  ***Sleep period:***  Overall r=0.29, p<0.001  Weekday r=0.27, p<0.001  Weekend r=0.09  ***Night wakings:***  Overall r=0.03  Weekday r=0.06  Weekend r=0.00 | Inadequate |
| Kushnir and Sadeh, 2013 [52] | N=30;  4-6 years;  14 female, 16 male;  Israel | **Brief Child Sleep Questionnaire (BCSQ)**  *Parental reported, referring to their child’s sleep during past week* | **Mini Motionlogger**  *Non-dominant wrist;*  *60 sec epoch;*  *Sadeh algorithm*^63^ | **Free living (no separate report of weekday/weekend day sleep- night time sleep only)**  Parents completed BSCQ based on their child’s sleep during past week, then child wore Mini Motionlogger, every night for 7 days. | **BCSQ:**   - Sleep time - Total sleep duration during the night - Number of night wakings - Total time awake during night   **Mini Motionlogger:**   - Sleep onset time - Sleep period - Number of night wakings (lasting ≥ 5 minutes) - Wake time after sleep onset | **Spearman’s Correlation:**  Sleep onset time = r=0.76, p<0.0001.  Sleep period r=0.85, p<0.0001  Night wakings r= 0.13  Wake after sleep onset r= -0.11  Parents underestimated sleep onset time and overestimated sleep period. Parents significantly underestimated the number of night wakings and wake after sleep onset. | Inadequate |
| Lucas-de la Cruz et al., 2016 [53] | N=51;  4-7 years;  48% female, 52% male;  Spain | **Child Sleep Habits Questionnaire (CSHQ)- Spanish version** | **Actisleep monitor**  *Non-dominant wrist;*  *60 sec epoch;*  *Nocturnal sleep/wake measures were estimated from actigraphic data using Sadeh algorithm*^63^*. Actigraph recordings were checked against the parent’s diary. The analysis period for the sleep data presented here (the scoring interval) included the period from the bedtime to the wake time as given in the diary.*  **Sleep diary** completed alongside accelerometer | **Free living (weekday/weekend day differences unknown)**  Child wore monitor on non-dominant wrist for 7 consecutive days (≥3 more nights to be included). Parents instructed to keep a log for time spent in bed, out of bed for each measured sleep episode. | **CSHQ:**   - Sleep latency - Total sleep time - Awakenings - Sleep efficiency   **Actisleep Monitor:**   - Sleep onset - Sleep latency - Total sleep time - Number and duration of awakenings - Sleep efficiency | **Polyserial correlation coefficient:**  Sleep latency (actisleep) with measures on CSHQ r=-0.318 to r= 0.355  Awakenings (actisleep) with measures on CSHQ r=-0.026 to r=0.334  Total sleep time (actisleep) with measures on CSHQ r=-0.154 to r=0.092  Efficency (actisleep) with measures on CSHQ r=0.105 to 0.133.  *ICC for ‘time in bed’ reported by CSHQ-S and sleep diary = 0.54, p<0.001 | Inadequate |
| Perpétuo et al., 2020 [54] | N=34;  3-6 years;  50% female, 50% male;  Portugal  Mothers' education level ranged from 6 to 21 years, and fathers from 6 to 17 years.  Mothers worked between 26 and 56 weekly hours and fathers worked between 35 and 60 hours per week. | **Children’s Sleep Habits Questionnaire (CSHQ)**  *Parental report on typical sleep* | **Actiwatch 2**  *Non-dominant wrist;*  *60 sec epoch;*  *Actiware 6.0.9 software was used to classify different sleep states.* | **Free living (week day and weekend day)**  Children asked to wear Actiwatch 2 for 7 days, questionnaire completed at one time point by parents.  Data on naps systematically collected. | **CSHQ:**   - Bed time - Wake up time - Daily sleep time - Number and duration of night wakings - Bedtime resistance - Sleep onset delay - Sleep duration - Night wakings - Parasomnia - Sleep apnea - Daytime sleepiness - Total sleep score   **Actiwatch 2**   - Bedtime - Wake up time - Time in bed - Total sleep time - Onset latency - Sleep efficiency - Wake after sleep onset - Number of night wakings | **Correlations**:  Total sleep time from actigraphy was correlated with CSHQ bedtime resistance (r = −0.43, p < 0.05) and sleep problem global score (r = −0.38, p < 0.05).  WASO (actigraphy) was significantly and positively correlated with CSHQ night-wakings (r = 0.44, p < 0.05).  More night-wakings registered by actigraphy were associated with parents' report of greater instability on sleep duration (r = 0.36, p < 0.05) and with more parasomnias (r = 0.35, p < 0.05).  Higher actigraphy sleep efficiency was significantly and negatively correlated with parent reports on bedtime resistance (r = −0.42, p < 0.05), night-wakings (r = −0.45, p < 0.01), and global scores of sleep problems (r = −0.45, p < 0.01).  **Bland Altman:**  With the exception of wake up time, there was no agreement between parental report and actigraphy measures  During the week, parents reported a mean of 7 min earlier (SD = 0:19 min) wake-up time compared to time registered by actigraphy. Difference between methods is higher for weekends, where parents reported wake-up time 28 min (SD = 0:47) later than the actigraph's.  For total sleep time and wake after sleep onset, the longer the child slept (and the longer the night-wakings lasted) the greater the difference between methods was (β = 0.49, p < 0.01 and β = −0.88, p < 0.001 respectively). Results showed that, for total sleep time, only 6.5% of the pairs of observations showed a difference of 30 min or less, and for 25.8% of the pairs of observations, the difference was of 1 h 30 or less. For WASO, only for 3% of the paired observations was the difference < 30 min; for 27%, the difference was < 1 hour. For all other variables, the difference was > 1 hour. | Inadequate |
| Sekine et al., 2002 [36] | N=21;  3-4 years old;  9 girls, 12 boys;  Japan | **Sleep habits questionnaire**  *3 consecutive nights of parental reported sleep* | **Actiwatch (MiniMitter)**  *Ankle of non-dominant leg;*  *60 sec epoch;*  *Device automatically estimates various sleep parameters according to the algorithm (after researcher entered bedtime and rising time)* | **Free living (weekday/weekend day differences unknown- night time sleep only)**  Questionnaire completed and Actiwatch worn for 3 consecutive days | **Sleep habits questionnaire:**   - Get up time - Bedtime - Sleeping hours   **Actiwatch**   - Sleep start - Time of sleep onset - Sleep end - The time of sleep termination - Assumed sleeping hours - Actual sleeping hours - Sleep latency - Sleep efficiency | **Pearson’s Correlation Coefficient and Paired t-tests:**  The correlation and difference  between the reported sleeping hours during the measurement  days and assumed or actual sleeping hours were 0.90 (p<0.001) and 0.79 hours (95%confidence interval: 0.59-0.99) and 0.90 (p<0.001) and 0.92 hours (0.73-1.10), respectively. The correlation  and difference between the assumed and actual sleeping  hours were 0.99 and 0.12 hours (0.06-0.19), respectively. | Doubtful |
| Werner et al., 2008 [37]  *also examined a sleep diary | N=50;  4.5 to 7.3 years;  44% female, 56% male;  Switzerland  Predominantly white.  50% middle class, 50% upper class. Lower socioeconomic class not represented. | **Sleep schedule time questionnaire (SSTQ)** | **Actiwatch Plus AW4**  *Non-dominant wrist;*  *60 sec epoch;*  *Sleep measures using Actiware 5, using scoring procedure described by Acebo et al. 2005^80^* | **Free living (week day and weekend day)**  SSTQ answered before an interview about child’s sleep that took place in parents’ homes, followed by child wearing Actiwatch and parental reported sleep diary data collected for 6-8 consecutive days (although 24 hour data collected, and SSTQ asked about nap times, there was no information available regarding day time sleep) | **Sleep schedule time questionnaire (SSTQ)**   - Normal wake up time - Get up time - Bedtime - Time of lights off - Sleep latency - Potential nap duration   **Actiwatch Plus AW4**   - Sleep start time - Sleep end time - Assumed sleep - Actual wake time - True sleep time | **Bland Altman and T-tests:**  **Actiwatch and SSTQ:**  A prior defined satisfactory agreement of 30 minutes was not reached for any parameters tested: sleep start, sleep end or assumed sleep.  **Sleep diary was also used:**  Sleep diary and SSTQ:  A prior defined satisfactory agreement of 30 minutes was not satisfied for any of the 3 parameters tested: sleep start, sleep end and assumed sleep, for weekdays or weekend days  No significant, sex, or socioeconomic status effects on the differences. | Inadequate |

***Diaries***

| **Study** | **Study sample** | **Measurement tool under study** | **Comparison measurement tool(s)** | **Methods** | **Outcome measures** | **Validity Results** | **Risk of bias rating** |
| --- | --- | --- | --- | --- | --- | --- | --- |
| Galland et al., 2016 [59] | N=74;  3.5 and 5 years;  Male to female ratio: 0:9 (3.5 yrs) and 1:3 (5 yrs);  New Zealand  Mostly European (98 % of 3.5 year olds; 91% 5 year olds) or Maori (2% 3.5 year olds ; 9% 5 of year olds)  Most children resided in neighbourhoods within mid-range of deprivation index (age 3.5 yrs = 4.9; 5 yrs= 4.4 | **Sleep diary**  *Parental reported (diary to be filled out by the person who is caring for the child during the measurement period- incl nursery teachers for day time naps)*  *2 days* | **Actical (MiniMitter)**  *Right hip*  *15 sec epoch*  *Data scored using automated script developed in MATLAB* | **Free living** (24 hour sleep – unknown if weekday / weekend day)  Children wore Actical for 5-7 days, sleep diary completed for 2 of these days. | **Sleep diary and Actical:**   - Sleep onset time - Sleep offset time - 24 hr sleep duration - Overnight sleep duration - Sleep efficiency - Night wakings (number and duration) - Naps (number and duration) | **Percent agreement:**  Agreement in nap time between the diary and Actical: 93%  **Kappa**:  Kappa for agreement in nap time between diary and Actical= 0.58  **Bland Altman**:  There were no significant systematic differences between any diary and Actical sleep–wake variables at ages 3½ or 5 years.  No significant differences in number of day time naps at any age.  Nap duration was significantly longer in data derived from diary compared to Actical in children 3½ years old.  No naps were identified in any child at age 5. | Doubtful |
| Iwasaki et al., 2010 [35]  *also examine a sleep questionnaire | N=47;  5 years old;  21 female, 27 male;  Japan | **Sleep diary**  *Parental reported daily sleep diary, when children stayed at nursery the sleep diary was recorded based on the information provided by their caretakers*. | **Ambulatory Monitoring**  *Wrist (dominant or non-dominant);*  *60 sec epoch;*  *Data analysed using Motion Logger ActFAST Analysis software, and sleep end time using Cole algorithm^69^* | **Free living (night time sleep only- weekend and weekend day reports separately)**  Ambulatory monitoring and sleep diary completed daily for 7 days, brief questionnaire completed at the end of the study period | **Sleep diary:**   - Sleep onset - Sleep end time - Sleep period - Number of night wakings (>5 minutes)   **Ambulatory Monitoring:**   - Bedtime - Sleep onset time - Sleep latency - Sleep end time - Sleep period - Number of night wakings | **Pearson’s Correlations:**  **Sleep diary and ambulatory monitoring:**  ***Sleep onset time***  Overall r=0.85, p<0.001  Weekday r=0.79, p<0.001  Weekend r=0.94, p<0.01  ***Sleep end time***  Overall r=0.83, p<0.001  Weekday r=0.81, p<0.001  Weekend r=0.70, p<0.001  ***Sleep period***  Overall r=0.57, p<0.001  Weekday r=0.43, p<0.001  Weekend r=0.73, p<0.001  ***Night wakings***  Overall r=0.02  Weekday r=0.01  Weekend r=0.03  **Comparison between questionnaire and diary:**  ***Sleep onset time:***  Overall r=0.38, p<0.001  Weekday r=0.45, p<0.001  Weekend r=0.07  ***Sleep end time:***  Overall r=0.53, p<0.001  Weekday r=0.64, p<0.001  Weekend r=0.18, p<0.01  ***Sleep period:***  Overall r=0.29, p<0.001  Weekday r=0.27, p<0.001  Weekend r=0.09  ***Night wakings:***  Overall r=0.03  Weekday r=0.06  Weekend r=0.00 | Inadequate |
| Jones and Ball, 2014 [60] | N=18;  3 years old;  Sex not reported;  England, UK  12 children from high socioeconomic status group and 6 from low socioeconomic status group | **Sleep diary**  *Parental reported during a typical week (when children were not ill or on holiday).* | **Actiwatch Mini**  *Placement and epoch not reported;*  *Proprietary software using medium sensitivity setting as recommended by user manual.* | **Free living** (24 hour sleep- weekday and weekend days)  Children wore Actiwatch and parent completed sleep diary for 5 consecutive days. Data on naps systematically collected. | **Sleep diary:**   - Bedtime - Sleep onset time - Morning wake time - Daytime nap duration - Sleep onset location   **Actiwatch:**   - Sleep onset time - Wake time - Daytime nap duration | **Paired sample t-tests and Pearson’s Correlations:**  Sleep onset time r=0.98, p<0.001  Wake time r=0.99, p<0.001  Nap duration r=0.81, p<0.001.  No systematic differences between families in low and high socioeconomic status groups. | Doubtful |
| Lam et al., 2011 [56] | N=59;  3-5 years;  30 female, 29 male;  USA  58% African American; 22% Caucasian; 5% Asian; 15% multiracial | **Parental sleep diary**  *Parents (with help of day care workers for nap times) instructed to maintain sleep diary for 7 days.* | **Motionlogger Actiwatch (MiniMitter)**  *Non-dominant wrist;*  *60 sec epoch;*  *Action W software program for sleep-wake cycles, Sadeh algorithm*^63^ *for sleep epochs* | **Free living (24 hour sleep – weekend and weekend day reports separately)**  Children wore Motionlogger Actiwatch and parents completed sleep diary for duration of 7 days. | **Parental sleep diary**   - Nap time - Bed time - Sleep latency - Night time awakenings - Sleep end time - Sleep onset time   **Motionlogger Actiwatch**   - Napping sleep onset and offset - Napping duration - Night time sleep onset and offset - Duration of night time sleep - Total weekday daytime napping - Total daytime weekend napping - Total weekday night time sleep - Total weekend night time sleep | **Paired t-tests:**  Parents significantly overestimated children’s night time sleep in comparison with Actiwatch, on both weekend and weekday nights.  Weekday and weekend nap duration did not differ significantly between diary and Actiwatch.  **ANOVA:**  Sleep onset times similar for parental report and Actiwatch (p=0.48)  Parents reported later sleep offset time than Actiwatch (p=0.34) (more so for the weekend than weekdays).  Parents reported fewer night time awakenings on all nights compared to Actiwatch (p<0.0001). | Inadequate |
| Tikotzky and Sadeh, 2001 [61] | N=59;  3.8 to 6.1 years;  30 female, 29 male;  Israel  Most children belonged to two parent families of a middle-upper socioeconomic status, highly educated and employed parents. | **Sleep diary**  *Parental reported daily sleep diary* | **AMA 32 Ambulatory Monitoring**  *Non-dominant wrist;*  *Epoch not reported;*  *Actigraphic Scoring Analysis program* | **Free living** (night time sleep only- weekdays only)  Child wore AMA 32 Ambulatory Monitoring device and, parent report child’s sleep in sleep diary for 4-5 consecutive nights | **Sleep diary:**   - Lights off time - Morning rise time - Sleep duration - Number of night wakings - Sleep quality - Duration to fall asleep - Evening sleepiness - Morning drowsiness - Sleep duration during the day   **AMA 32 Ambulatory Monitoring**  Sleep schedule measures:   - Sleep onset time - Morning rising time - Total sleep duration   Sleep quality measures:   - Sleep percent (sleep efficiency) - True sleep time - Number of night wakings | **Pearson’s Correlations and t-tests:**  Lights off time (parental report) and sleep onset time (accel) r=0.87, p<.0005  Sleep latency (parental report) and sleep onset (accel) r=0.02  Morning rise time from both measures r=0.87, p<0.0005  Sleep duration from both measures r=0.81, p<0.0005  Night wakings from both measures r=0.42, p<0.005. Significant difference between average n of night wakings according to parental (0.45) and accelerometer (2.66), p<0.001.  Number of night wakings explained 86% of variance in the difference between parental and accelerometer reports on night wakings, age of child explain an additional 2.6% of the variance.  Sleep quality (parental report) and sleep efficiency (accel) r=-0.28, p<0.05  Sleep quality (parental report) and true sleep time r=-0.12  Sleep quality (parental report) and night time wakings r=0.35, p<0.01 | Very good |
| Werner et al., 2008 [37]  * also examine a sleep questionnaire | N=50;  4.5 to 7.3 years;  44% female, 56% male;  Switzerland  Predominantly white;  50% middle class, 50% upper class. Lower class not represented. | **Sleep diary**  *Parental reported sleep diary* | **Actiwatch Plus AW4**  *Non-dominant wrist;*  *60 sec epoch;*  *Sleep measures using Actiware 5, using scoring procedure described by Acebo et al. 2005^80^* | **Free living (week day and weekend day)**  SSTQ answered before an interview about child’s sleep that took place in parents’ homes, followed by child wearing Actiwatch and parental reported sleep diary data collected for 6-8 consecutive days (although 24 hour data collected, and SSTQ asked about nap times, there was no information available regarding day time sleep) | **Sleep diary**   - Sleep start - Sleep end - Assumed sleep - Nocturnal wake time - Actual sleep time   **Actiwatch Plus AW4**   - Sleep start time - Sleep end time - Assumed sleep - Actual wake time - True sleep time | **Bland Altman and T-tests:**  **Sleep diary and Actiwatch**  A priori, satisfactory agreement= differences > 30 minutes. This requirement was satisfied for 3 of our 5 parameters: sleep start (±28 , sleep end (±24) and assumed sleep (±32).  This requirement was not satisfied for actual sleep time (±72) and nocturnal wake time (±55). For weekdays and weekend days, the differences were larger.  **Questionnaire was also used:**  Sleep diary and SSTQ:  A prior defined satisfactory agreement of 30 minutes was not satisfied for any of the 3 parameters tested: sleep start, sleep end and assumed sleep, for weekdays or weekend days.  No significant age, sex, or socioeconomic effects on any of these differences. | Inadequate |

**Supplementary file 8: Reliability of measurement tools used to assess sleep of pre-school aged children.**

T***est-retest reliability of questionnaires used to measure sleep of pre-school aged children***

| **Study** | **Study Sample** | **Measurement tool under study (for reliability)** | **Methods** | **Test-retest results** | **Risk of bias rating** |
| --- | --- | --- | --- | --- | --- |
| Ishihara et al., 2014 [51] | Japan;  N=346;  3-6 years;  158 female, 188 male | Children's ChronoType Questionnaire (CCTQ)- Japanese version | Fill out the questionnaire two weeks apart. | **Pearson’s Correlation coefficient:**  r=0.90 (p<0.001) | Inadequate |
| Lucas-de la Cruz et al., 2016 [53] | Spain;  N=52;  4-7 years;  Sex of participants unknown | Child Sleep Habits Questionnaire (CSHQ)- Spanish version | Questionnaire completed twice in a three week interval. | **Intraclass correlation coefficient**  Bedtime resistance: 0.75, p<0.001  Sleep duration 0.78, p<0.001  Sleep anxiety 0.79, p<0.001  Night wakings 0.56, p<0.001  Parasomnias 0.73, p<0.001  Sleep disordered breathing 0.76, p<0.001  Daytime sleepiness 0.54, p<0.001  **Pearson’s correlation coefficient**  Bedtime resistance: 0.75, p<0.001  Sleep onset delay 0.69  Sleep duration 0.81, p<0.001  Sleep anxiety 0.79, p<0.001  Night wakings 0.56, p<0.001  Parasomnias 0.74, p<0.001  Sleep disordered breathing 0.77, p<0.001  Daytime sleepiness 0.60, p<0.001 | Inadequate |

***Internal consistency of measurement tools used to assess sleep of pre-school aged children***

| **Study** | **Study Sample** | **Measurement tool under study (for reliability)** | **Methods** | **Internal consistency results** | **Level of evidence (RoB)** |
| --- | --- | --- | --- | --- | --- |
| Ishihara et al., 2014 [51] | Japan;  N=346;  3-6 years;  158 female, 188 male | Children's ChronoType Questionnaire (CCTQ)- Japanese version | Parents completed the Japanese version of the CCTQ | **Cronbach’s alpha (α)**  Overall scale = 0.77, individual items ranged from 0.72 to 0.76. | Very good |
| Lucas-de la Cruz et al., 2016 [53] | Spain;  N=286;  4-7 years; | Child Sleep Habits Questionnaire (CSHQ)- Spanish version | Parents completed the Child Sleep Habits Questionnaire (CSHQ)- Spanish version | **Cronbach’s alpha (α)**  Overall scale = 0.81.  Bedtime resistance = 0.65  Sleep duration = 0.81  Sleep anxiety =0.74  Night wakings = 0.69  Parasomnias = 0.62  Sleep disordered breathing = 0.66  Daytime sleepiness = 0.60 | Very good |
| Tan et al., 2018 [55] | N=171;  4-5 years; 53% female, 47% male;  China  Chinese kindergarteners  Most parents worked full-time. Over 80% of mothers and 85% fathers had at least a college level education. Over half of the mothers and 80% fathers made 5000 or more monthly. | Children’s Sleep Habits Questionnaire (CSHQ)- Chinese version | Parents completed the Chinese version of the CSHQ-C | **Cronbach’s alpha (α)**  Overall scale = 0.67  Bedtime resistance = 0.59  Daytime sleepiness = 0.62  Night wakings = 0.11  Parasominas = 0.58  Sleep anxiety = 0.54  Sleep disordered breathing= 0.36  Sleep duration = 0.65 | Very good |
| Staples et al., 2019 [58] | N=107 (at 3 years), n=104 (at 3.5 years);  3-3.5 years;  Sex not reported for this age category;  USA  Information on parental education, ethnicity and socioeconomic status was available but for the whole sample, not separated for age groups of interest. Predominantly college educated parents, European American, high socioeconomic status. | MicroMini Motionlogger Accelerometer;  *Wrist worn;*  *Scored using Action W-2 software and Sadeh algorithm^63^*  *Sleep diary also completed to record bedtime, night awakenings, rise times and other issues relating to child’s sleep.* | Accelerometer worn for 7 days at three time points in longitudinal study (2.5 years, 3 years, 3.5 years). Principal component analysis to determine composites. | **Cronbach’s alpha (α)**  Sleep activity  Aged 3 years: 0.93  Aged 3.5 years: 0.93  Sleep variability  Aged 3 years: 0.89  Aged 3.5 years: 0.90  Sleep timing  Aged 3 years: 0.95  Aged 3.5 years: 0.95  Sleep duration  Aged 3 years: 0.90  Aged 3.5 years: 0.92 | Very good |

**Supplementary file 9: Feasibility assessment of measurement tools included in the review**

| **Study** | **Study Sample** | **Measurement tool**  **(*Measurement instrument under study or comparison tool*)** | **Feasibility information** |
| --- | --- | --- | --- |
| Duraccio et al., 2017 [34] | N=108;  4-6 years;  42% female; 53% male; 5% missing  USA  86% Caucasian, 2% Hispanic, >1% Native American, 2% Asian, 5% Other, 5% Missing  Mean monthly income = $5,693.73  (Likely high socioeconomic profiles as recruited through universities) | Accelerometer- Actigraph GT3X+ (***comparison tool***) | 3% missing data due to participants missing 2 (out of 3) nights of data. |
| Ishihara et al., 2014 [51] | N=72 (validity), n=346 (reliability);  3-6 years;  37 female, 35 male;  (validity), 158 female, 188 male (reliability);  Japan | Children's ChronoType Questionnaire (CCTQ)- Japanese version *(****measurement instrument under study)*** | 3% missing data on test-retest due to missing at least one question on the questionnaire |
|  |  | Accelerometer- Actiwatch 2 (***comparison tool***) | 4% missing from validation study due to missing data on accelerometer from participant non-adherence |
| Iwasaki et al., 2010 [35] | N=47;  5 years old;  21 female, 27 male;  Japan | Accelerometer -Ambulatory monitoring (***comparison tool***) | 1 child (out of 48 recruited) excluded prior to the study due to still taking day time naps.  Actigraphy ultimately tolerated by all participants- data successfully collected for all children included in study.  Cost of devices were expensive: Accelerometers costs more than US$25,000 to introduce a system with licensed software and 15 devices. High cost resulting in more likely use of questionnaire/diary based sleep assessment tools |
| Jones and Ball, 2014 [60] | N=18;  3 years old;  Sex not reported;  England, UK  12 children from high socioeconomic status group and 6 from low socioeconomic status group | Accelerometer -Actiwatch (***comparison tool***) | 49% missing data due to (17 out of 35) children refusing to keep Actiwatch on. |
| Kushnir and Sadeh, 2013 [52] | N=30;  4-6 years;  14 female, 16 male;  Israel | Accelerometer -Mini Motionlogger (***comparison tool***) | Compliance problems and technical issues reported- actigraphy data were available for ≥ 4 nights in 88% percent of the children. For the rest of the sample, actigraphy data were available in 4.5%, 3%, and 4.5% for 1, 2, or 3 nights, respectively. However, it’s not possible to distinguish between whether these reports were related to clinical or healthy sample of children. |
| Lam et al., 2011 [56] | N=59;  3-5 years;  30 female, 29 male;  USA  58% African American; 22% Caucasian; 5% Asian; 15% multiracial | Accelerometer -Motionlogger Actiwatch (***comparison tool***) | 8% missing data due to noncompliance with accelerometer (=5 children dropped out of the study due to noncompliance with accelerometer). |
| Lucas-de la Cruz et al., 2016 [53] | N=51 (validity);  4-7 years;  48% female, 52% male;  Spain | Children’s Sleep Habits Questionnaire (CSHQ)-Spanish version *(****Measurement instrument under study)*** | 40% did not complete CSHQ correctly on first administration  Completion of questionnaire takes 4-6 minutes |
|  |  | Accelerometer -ActiSleep (***comparison tool***) | 33% did not wear accelerometer for required time |
|  |  | Both tools (***Measurement instrument under study and comparison tool***) | 16% excluded from validity analysis due to not completing all measures (CSHQ, ActiSleep, sleep diary) - 61 parents completed second administration, of which 51 had appropriate data for all three measures. |
| Meltzer et al., 2015 [50] | N=14  3-5 years;  Sex not reported for this age category;  USA  Ethnicity reported for whole sample, but not separately by age category. | Fitbit Ultra *(****measurement instrument under study)*** | 19% missing data due to technical issues with Fitbit Ultra.  However, it’s not possible to distinguish between the different age groups for the missing data (full sample = children aged 3-17 years) |
|  |  | Polysomnography (***comparison tool***) | 3% missing data due to corrupt polysomnography files  However, it’s not possible to distinguish between the different age groups for the missing data (full sample = children aged 3-17 years) |
| Perpétuo et al., 2020 [54] | N=34;  3-6 years;  50% female, 50% male;  Portugal  Mothers' education level ranged from 6 to 21 years, and fathers from 6 to 17 years.  Mothers worked between 26 and 56 weekly hours and fathers worked between 35 and 60 hours per week. | Children’s Sleep Habits Questionnaire (***comparison tool***) | 20% missing data due to non-completion |
|  |  | Accelerometer -Actiwatch 2 (***comparison tool***) | 11% missing data due to not having valid data |
|  |  | Both tools (***Measurement instrument under study and comparison tool***) | 26% participants had missing data for both measurement tools  Prior to measurement- 17% families dropped out for various reasons (changed residence or withdrew consent). |
| Sekine et al., 2002 [36] | N=21;  3-4 years old;  9 girls, 12 boys;  Japan | Accelerometer -Actiwatch ***(comparison tool)*** | 12% missing data from accelerometer (1 child reluctant to wear instrument, data from 2 other children were not successfully measured). |
| Staples et al., 2019 [58] | N=107 (at 3 years), n=104 (at 4.5 years);  3-3.5 years;  Sex not reported for this age category;  USA  Information on parental education, ethnicity and socioeconomic status was available but for the whole sample, not separated for age groups of interest. Predominantly college educated parents, European American, high socioeconomic status. | Accelerometer -MicroMini Motionlogger *(****measurement instrument under study)*** | At age 3 years:  26% missing data (planned missingness)  5% accelerometer failure  6% not available to participate  At age 3.5 years:  18% missing data (planned missingness)  7% accelerometer failure  4% not available to participate |
| Werner et al., 2008 [37] | N=50;  4.5 to 7.3 years;  44% female, 56% male;  Switzerland  Predominantly white.  50% middle class, 50% upper class socioeconomic status. Lower socioeconomic status class not represented. | Accelerometer -Actiwatch Plus AW4 ***(comparison tool)*** | 26% total missing data: 13% insufficient language skills/deregistration, remaining 13% accelerometer problems, including: 4% refusal to wear accelerometer; 6% technical problems; 3% accelerometer loss |

**Supplementary File 10: Sub-analysis based on children aged 3-5 years old only.**

## Results

### Study selection and characteristics

Of the sixteen studies included in this review, eight included children aged 3-5 years only. In this supplementary file, we will discuss the results of these eight studies only.

Included articles were published between 2002 and 2019, and were all conducted in high income countries (49): USA (n=3), Japan (n=2), China (n=1), New Zealand (n=1), and UK (n=1). Sample sizes ranged from 14 (50) to 171 children (55). All studies that reported the sex of the children included both male and female children. Studies examined the measurement properties of the tools in free-living conditions to determine habitual sleep behaviours (n=7). One study was laboratory based and used polysomnography as the criterion method to measure sleep (50).

Six studies examined parental reported measurement tools (questionnaires and diaries), and two remaining studies examined accelerometers. Seven studies examined the measurement properties of one measurement tool only and one study examined the measurement properties of two measurement tools (35). Three studies examined questionnaires; one study generated a new questionnaire (36), one adapted a questionnaire developed for other age groups (35), and one used a Chinese translated version of the *Children’s Sleep Habits Questionnaire* (CSHQ) a measure originally developed for children aged 4-10 years old (55). Four studies examined the measurement properties of different diaries. Diaries were specific to the study, with one study utilising parental diaries frequently used within a clinical care setting (56). Two studies examined the measurement properties of two types of accelerometer: Fitbit Ultra and MicroMini Motionlogger.

The most frequently reported measurement property was convergent validity (n=5). Two studies examined the construct (structural) validity of the tools, one examined criterion validity, and two the internal consistency. No studies reported the qualitative feasibility of the tools, though six studies reported missing data or non-completion that demonstrated an element of feasibility.

### Risk of bias

The two studies assessing construct (structural) validity were quality assessed as adequate (55, 58). One study assessing criterion validity was quality assessed as inadequate (50) due to the type of analysis conducted. The majority of studies assessing the convergent validity of the tools were quality assessed as high risk of bias; inadequate (35, 56) and doubtful (36, 59-60). Main reasons for poor methodological quality of the studies was due to unknown measurement properties of the comparator tools. Two studies examining internal consistency were quality assessed as very good (55, 58). The full risk of bias assessment can be found in supplementary file 4. No studies were removed from the overall analysis based on the risk of bias assessment, however, it must be noted that in this sub-analysis, only the studies assessing construct validity and internal consistency were deemed to be of low risk of bias.

### Summary of measurement properties of measurement tools

### *Validity*

#### Construct (structural) Validity

For children aged up to 5 years old, only one study examined the construct validity of the Chinese translation of the CSHQ (55). Tan and colleagues conducted a confirmatory factor analysis and determined that no existing factor structures were suitable for the data with pre-school aged children. Following this, they performed an exploratory and a further confirmatory factor analysis to determine a new four factor structure, including: bedtime behaviours, sleep behaviours, morning waking, and daytime sleepiness (55).

One study examined the construct validity of the outcome measures of the MicroMini Motionlogger accelerometer using principal component analysis (58). This study revealed a four component structure: sleep activity, sleep variability, sleep timing and sleep duration, which could categorise accelerometer outcome variables. Daytime sleep and sleep latency represented exclusive elements of sleep that could not be categorised into factor structures.

#### Criterion Validity

One study examined the criterion validity of the Fitbit Ultra compared with polysomnography (50). The Fitbit Ultra (sensitive mode) underestimated sleep time and sleep efficiency, and overestimated wake after sleep onset. The Fitbit Ultra (normal mode) overestimated sleep time and sleep efficiency, but there was no significant difference for wake after sleep onset.

#### Convergent Validity

***Accelerometers***

No studies examined the convergent validity of accelerometers in children aged 3-5 years old only.

***Questionnaires***

For children aged 3-5 years old, two studies examined the convergent validity of two different parental reported questionnaires. The recall periods of the questionnaires included daily reporting (36), and the past month (35). Neither questionnaires systematically collected data on naps (35-36). One questionnaire examined weekday and weekend days separately (35).

Both questionnaires assessed sleep duration. The *sleep habits questionnaire* (36) showed high convergence with accelerometers for determining sleep duration (r=0.85 to r=0.90). The *brief 12 item questionnaire* showed weak associations with accelerometry for determining sleep duration, sleep latency, night awakenings, bed time, sleep onset time and wake up time (35).

***Diaries***

Four studies examined the convergent validity of sleep diaries. The format of the diaries varied, some used time intervals to determine sleep/wake status (56, 59), whilst others requested certain information, such as sleep onset and end time (35, 60). All diaries were parental reported, however, three studies explicitly stated that day time sleep records were based on information from nursery teachers (35, 56, 59).

Diaries were completed daily in all studies (35, 56, 59, 60), for a duration of 2 (59) to 7 days (35, 56). The majority of diaries collected data on nap times (37, 56, 59, 60), however only three reported results on this outcome measure (56, 59, 60). Two studies reported results for weekday and weekend days separately (35, 56). All studies used an accelerometer as the comparison tool; different types of accelerometers were used. Diary and accelerometer data were collected simultaneously in all studies.

Three studies demonstrated that the sleep diaries were highly convergent for determining nap times in comparison with accelerometry (56, 59, 60). Diaries were generally similar to accelerometry for determining sleep onset (35, 56, 60) and sleep end/wake time (35, 60), with the exception of one study where parents reported that the child rose later than that detected by the accelerometer (56). Diaries reporting sleep duration showed weak associations with accelerometry (56), and weak correlations for weekday but moderate for weekend days (35). Similar to questionnaires, diaries were rated as weak for determining night awakenings (35, 56).

### *Reliability*

#### Test-retest

No studies examined the test-retest reliability of any measurement tool in children aged 3-5 years old only.

#### Internal Consistency

One study showed moderate internal consistency of the Chinese version of the CSHQ, (α=0.67) (55).

The newly devised four factor structure for the MicroMini Motionlogger accelerometer (sleep activity, sleep variability, sleep timing and sleep duration, which could categorise accelerometer outcome variables) were determined to have high internal consistency (ranging from α =0.89 to 0.95) (58).

### *Feasibility*

No studies explicitly examined the feasibility of the measurement tools through qualitative research. However, as per previous reviews (39), we included missing data, non-completion and other indicators of feasibility of the measure (such as completion time). Feasibility information primarily consisted of missing data from the accelerometer, either when used as the comparison tool (36, 56, 60) or tool under study (50, 58) for reasons including: technical problems, refusal to wear the device, or a lack of available valid data. One study reported that although the accelerometers were tolerated by the children, the cost of such devices (and associated licensed software required) was expensive (35).

### Generalisability of results

***Ethnicity***

Studies that reported the ethnicity of the included children had samples who were predominantly Caucasian (59, 60), Chinese (55), and African American children (56).

***Socioeconomic profile***

Studies that reported the socioeconomic profile of participants described the families of the children as mainly middle-upper class (60), and one study reported that children lived in areas within ‘mid-range’ of deprivation (59). One study reported the educational level and working hours of parents of the included children (55). The one study reporting children mainly as middle-upper class described no systematic differences in the outcomes of the diary and accelerometry between families in low and high socioeconomic groups (60).

References in supplementary files, not included in main manuscript:

79. Choi L, Liu Z, Matthews CE, Buchowski MS. Validation of accelerometer wear and nonwear time classification algorithm. Medicine and science in sports and exercise. 2011;43(2):357-364. doi:10.1249/MSS.0b013e3181ed61a3

80. Acebo C, Sadeh A, Seifer R, Tzischinsky O, Hafer A, Carskadon MA. Sleep/Wake Patterns Derived from Activity Monitoring and Maternal Report for Healthy 1- to 5-Year-Old Children. Sleep. 2005;28(12):1568-1577. doi:10.1093/sleep/28.12.1568
